# Androgen receptor splice variant-7 expression emerges with castration resistance in prostate cancer

**DOI:** 10.1172/JCI122819

**Published:** 2018-11-26

**Authors:** Adam Sharp, Ilsa Coleman, Wei Yuan, Cynthia Sprenger, David Dolling, Daniel Nava Rodrigues, Joshua W. Russo, Ines Figueiredo, Claudia Bertan, George Seed, Ruth Riisnaes, Takuma Uo, Antje Neeb, Jonathan Welti, Colm Morrissey, Suzanne Carreira, Jun Luo, Peter S. Nelson, Steven P. Balk, Lawrence D. True, Johann S. de Bono, Stephen R. Plymate

**Affiliations:** 1 The Institute of Cancer Research, London, United Kingdom.; 2 The Royal Marsden, London, United Kingdom.; 3 Fred Hutchinson Cancer Research Center, Seattle, Washington, USA.; 4 Department of Medicine, University of Washington, Seattle, Washington, USA.; 5 Beth Israel Deaconess Medical Center, Boston, Massachusetts, USA.; 6 Johns Hopkins University School of Medicine, Baltimore, Maryland, USA.; 7 Puget Sound VA Health Care System, Geriatric Research Education and Clinical Center (PSVAHCS-GRECC), Seattle, Washington, USA.

**Keywords:** Oncology, Prostate cancer

## Abstract

**BACKGROUND.** Liquid biopsies have demonstrated that the constitutively active androgen receptor splice variant-7 (AR-V7) associates with reduced response and overall survival from endocrine therapies in castration-resistant prostate cancer (CRPC). However, these studies provide little information pertaining to AR-V7 expression in prostate cancer (PC) tissue.

**METHODS.** Following generation and validation of a potentially novel AR-V7 antibody for IHC, AR-V7 protein expression was determined for 358 primary prostate samples and 293 metastatic biopsies. Associations with disease progression, full-length androgen receptor (AR-FL) expression, response to therapy, and gene expression were determined.

**RESULTS.** We demonstrated that AR-V7 protein is rarely expressed (<1%) in primary PC but is frequently detected (75% of cases) following androgen deprivation therapy, with further significant (**P** = 0.020) increase in expression following abiraterone acetate or enzalutamide therapy. In CRPC, AR-V7 expression is predominantly (94% of cases) nuclear and correlates with AR-FL expression (**P** ≤ 0.001) and AR copy number (**P** = 0.026). However, dissociation of expression was observed, suggesting that mRNA splicing remains crucial for AR-V7 generation. AR-V7 expression was heterogeneous between different metastases from a patient, although AR-V7 expression was similar within a metastasis. Moreover, AR-V7 expression correlated with a unique 59-gene signature in CRPC, including HOXB13, a critical coregulator of AR-V7 function. Finally, AR-V7–negative disease associated with better prostate-specific antigen (PSA) responses (100% vs. 54%, **P** = 0.03) and overall survival (74.3 vs. 25.2 months, hazard ratio 0.23 [0.07–0.79], **P** = 0.02) from endocrine therapies (pre-chemotherapy).

**CONCLUSION.** This study provides impetus to develop therapies that abrogate AR-V7 signaling to improve our understanding of AR-V7 biology and to confirm the clinical significance of AR-V7.

**FUNDING.** Work at the University of Washington and in the Plymate and Nelson laboratories is supported by the Department of Defense Prostate Cancer Research Program (W81XWH-14-2-0183, W81XWH-12-PCRP-TIA, W81XWH-15-1-0430, and W81XWH-13-2-0070), the Pacific Northwest Prostate Cancer SPORE (P50CA97186), the Institute for Prostate Cancer Research, the Veterans Affairs Research Program, the NIH/National Cancer Institute (P01CA163227), and the Prostate Cancer Foundation. Work in the de Bono laboratory was supported by funding from the Movember Foundation/Prostate Cancer UK (CEO13-2-002), the US Department of Defense (W81XWH-13-2-0093), the Prostate Cancer Foundation (20131017 and 20131017-1), Stand Up To Cancer (SU2C-AACR-DT0712), Cancer Research UK (CRM108X-A25144), and the UK Department of Health through an Experimental Cancer Medicine Centre grant (ECMC-CRM064X).

## Introduction

Prostate cancer (PC) is the most commonly diagnosed noncutaneous cancer and the second leading cause of male cancer-related death in the Western world (1). Androgen receptor (AR) signaling is critical for PC development and progression (2–5). PC patients with advanced disease after primary therapy respond robustly to androgen deprivation therapy (ADT), but nearly all will progress to fatal castration-resistant PC (CRPC). There is now mounting evidence that progression to CRPC remains dependent on persistent AR signaling driven by increased androgen synthesis, overexpression of AR coactivators, AR amplification, and AR-activating point mutations (3, 5–9). These molecular findings have driven the development of new anti-androgen therapies, such as abiraterone acetate (AA), enzalutamide (E), and apalutamide, that target the AR axis in patients with castration-sensitive PC (CSPC) and CRPC. These therapies have led to improved patient outcomes and health-related quality of life (10–18).

Despite this significant progress, resistance to AA and E is common and on average occurs within a year of starting therapy; this is due, at least in part, to the emergence of constitutively active AR splice variants, of which AR splice variant-7 (AR-V7) is regarded as the most significant and most extensively characterized (19–31). AR-V7 is thought to arise from aberrant mRNA splicing of AR exons 1–3, loss of exons 4–8, and inclusion of cryptic exon 3 (CE3) into the transcribed AR gene (22, 31). The resultant protein product is constitutively active in the absence of androgens and drives growth of PC cell lines and patient-derived xenografts in the presence of AR-directed therapies such as AA or E (23, 24, 31, 32). AR-V7 forms homodimers with itself and heterodimers with full-length AR (AR-FL) and in the absence of androgens binds to AR response elements, facilitating the generation of a protumorigenic transcriptome (33). In addition, transgenic mice with forced expression of AR-V7 display a protumorigenic phenotype (34). These preclinical studies confirm that AR-V7 may facilitate ligand-independent AR signaling to drive resistance to established endocrine therapies.

Insufficient data have been available on AR-V7 mRNA and protein expression in primary PC, although some studies suggest expression (35–38). AR-V7 protein expression increases as patients develop CRPC and resistance to AA or E (29–31, 39). A plethora of clinical studies have confirmed AR-V7 expression to be correlated with resistance to AA and E therapy in CRPC; the majority of these measured AR-V7 mRNA or protein from liquid biopsies (i.e., circulating tumor cells, exosomes, or whole blood) as opposed to using metastatic tumor biopsies (19, 20, 26–30, 40–42). All clinically licensed therapies modulate AR activity through its ligand-binding domain and therefore conceptually have no activity against AR-V7–mediated oncogenic signaling. Pharmacological inhibitors of bromodomain and extraterminal proteins and HSP90 suppress AR-V7 generation through inhibition of mRNA splicing and inhibit the growth of CRPC models (32, 43, 44). However, these therapies target multiple cellular pathways, and therefore concerns with regard to clinical utility and safety remain. The development of novel therapies that overcome AR-V7 signaling in CRPC remains an area of urgent unmet clinical need.

In this work, we have performed an extensive cross-institutional study to determine nuclear AR-V7 protein expression in tissue biopsies and autopsies from primary and metastatic PC tumors using a potentially novel AR-V7 antibody. We establish that expression of AR-V7 protein is rare in primary PC. In addition, nuclear AR-V7 expression emerges in response to primary ADT alone in most patients, and further increases in response to AA or E therapy, with nuclear AR-V7 being an important marker of response to these endocrine therapies in CRPC. Furthermore, AR-V7 expression associates with AR-FL expression and AR copy number in CRPC, although many cases with high AR-FL protein expression have undetectable/low AR-V7 protein expression. Moreover, nuclear AR-V7 expression is heterogeneous in different CRPC metastases in the same patient. Finally, nuclear AR-V7 expression is associated with a unique gene signature in CRPC patients. These data support a critical role for AR-V7 in CRPC biology and resistance to established endocrine therapies, providing further impetus for the development of therapeutic strategies that overcome AR-V7–mediated signaling to improve the outcome for patients with this lethal disease.

## Results

### Validation and optimization of a AR-V7 antibody (Clone RM7) for immunohistochemistry.

**Figure 1:**
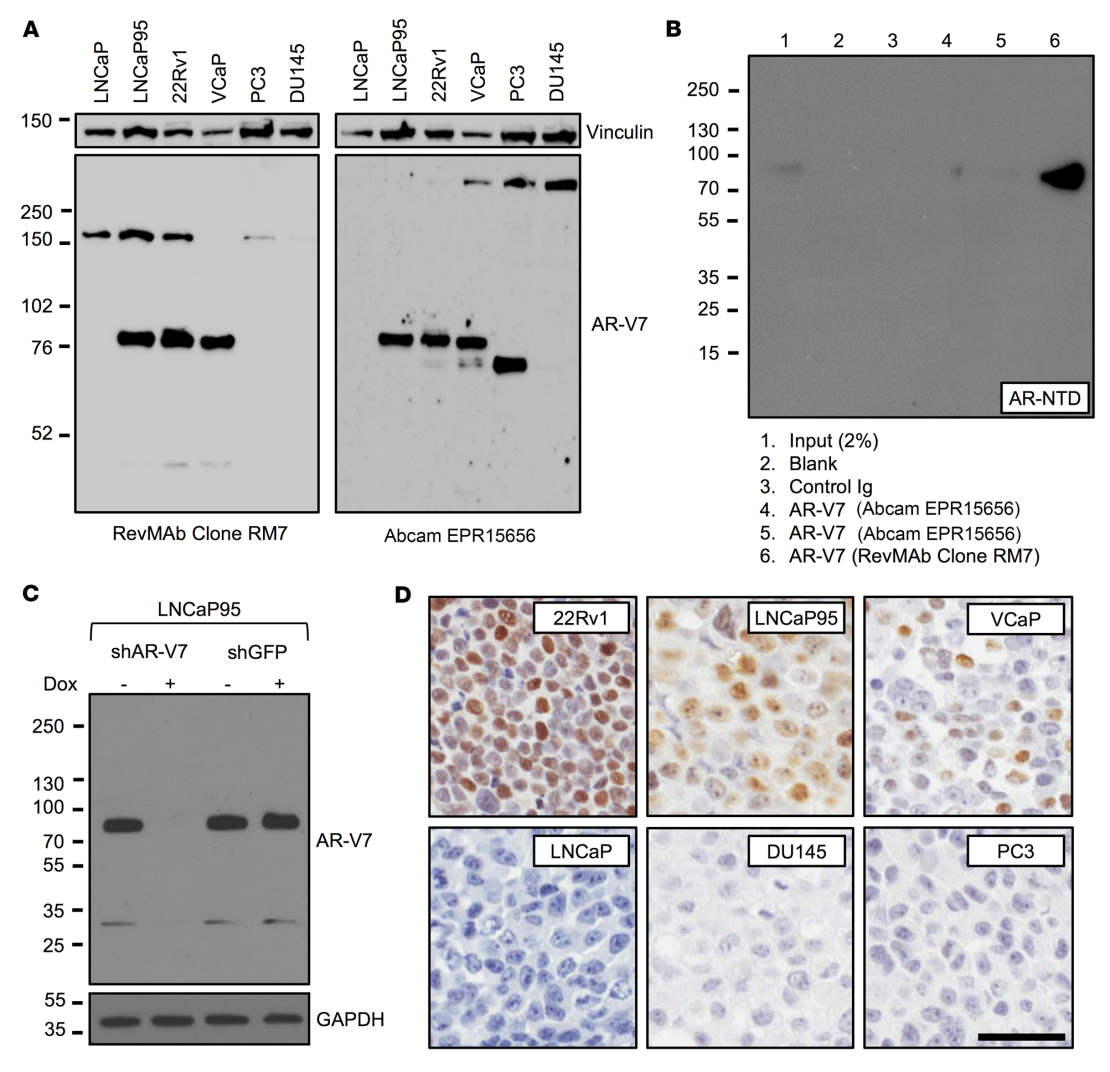
Validation and optimization of a potentially novel AR-V7 antibody (Clone RM7) for immunohistochemistry. (**A**) Western blot (long exposure) of AR-V7–positive (LNCaP95, 22Rv1, and VCaP) and –negative (LNCaP, PC3, and DU145) PC cell lines using a novel recombinant rabbit monoclonal anti–AR-V7 antibody (Clone RM7) and a previously reported anti–AR-V7 antibody (EPR15656). All cell lines except LNCaP95 (10% charcoal-stripped serum) were grown in 10% FBS. (**B**) Immunoprecipitation of AR-V7 from M12–cumate-inducible AR-V7 cells using the same concentration of AR-V7 antibodies and Western blot performed with AR N-terminal domain (AR-NTD) antibody. (**C**) LNCaP95 cells with doxycycline-inducible shRNA to AR-V7 were treated with (or without) doxycycline and Western blot performed with AR-V7 antibody (RM7). (**D**) Micrographs of AR-V7 detection by IHC using AR-V7 antibody (RM7) in cell line pellets positive (22Rv1, LNCaP95, and VCaP) and negative (LNCaP, DU145, and PC3) for AR-V7 (original magnification, ×200; scale bar: 50 μm).

**Figure 2:**
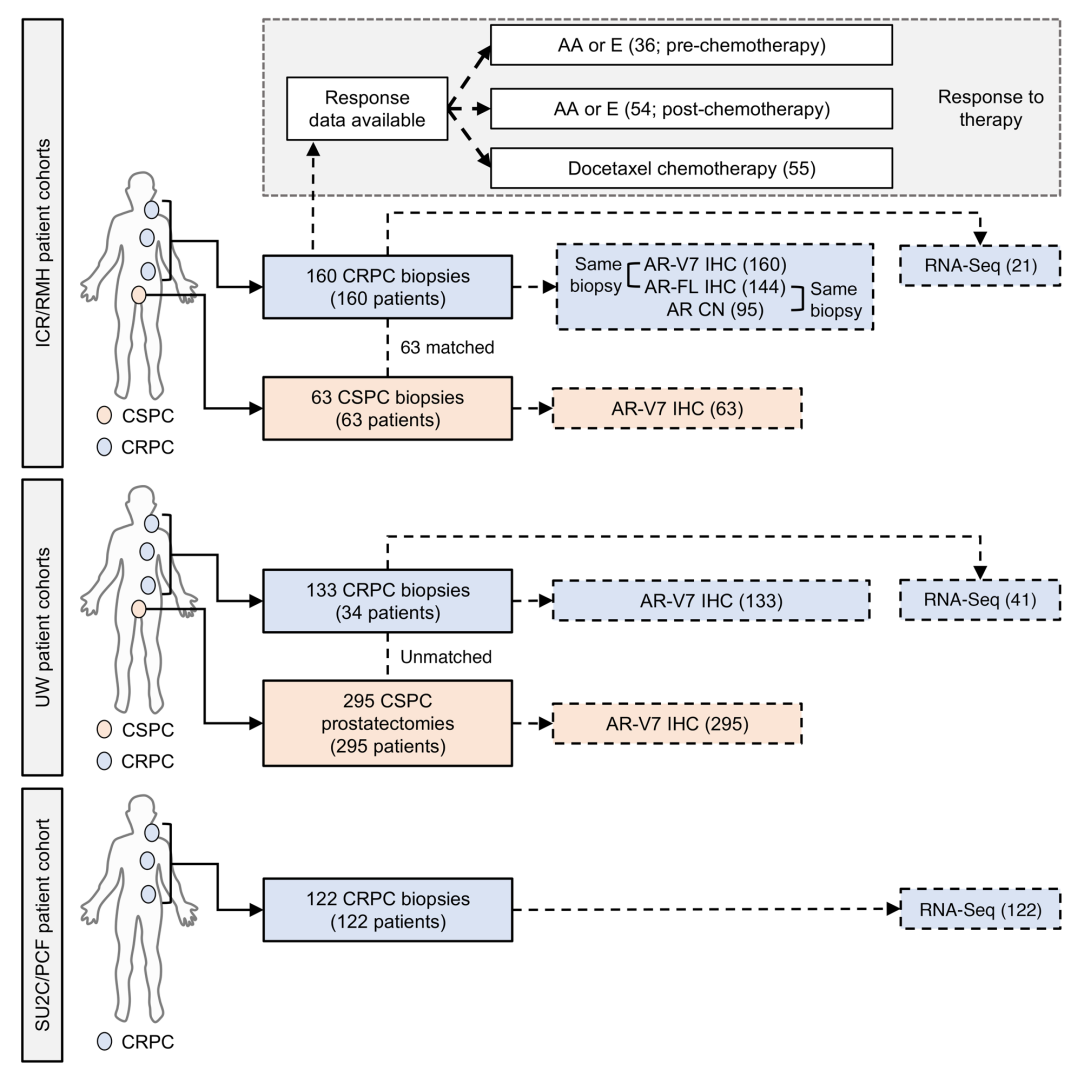
Summary of clinical samples analyzed. Overview of the ICR/RMH, UW, and SU2C/PCF patient cohorts utilized for this study. The ICR/RMH patient cohort included 63 CSPC biopsies and 160 CRPC biopsies stained for nuclear AR-V7 expression. Of the 160 biopsies with AR-V7 expression, AR-FL expression was available in 144 biopsies, AR copy number in 95, and RNA-Seq in 21. Response data were available for AA or E before chemotherapy in 36 patients, AA or E after chemotherapy in 54 patients, and docetaxel chemotherapy in 55 patients. The UW patient cohort included 295 CSPC tissues (from 295 patients) who had radical prostatectomy as primary therapy and 133 CRPC biopsies of metastases (from 34 patients). Of 133 CRPC biopsies from 34 patients with AR-V7 expression, RNA-Seq was available in 41 biopsies. The SU2C/PCF patient cohort included 122 CRPC biopsies with RNA-Seq analysis.

**Figure 3:**
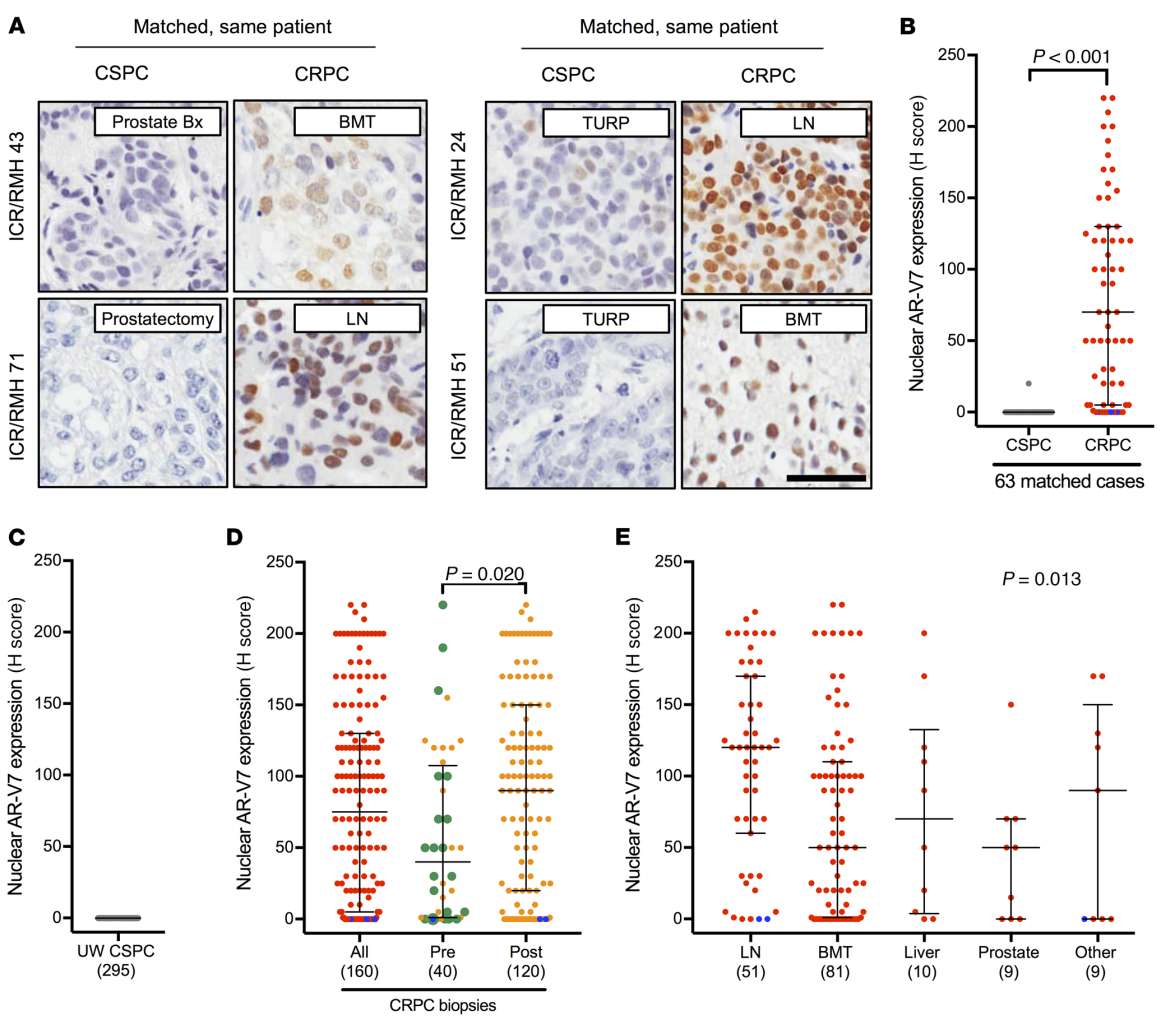
AR-V7 protein expression in PC. (**A**) Representative micrographs of AR-V7 detection by IHC in 4 ICR/RMH patients with matched CSPC and CRPC biopsies (original magnification, ×200; scale bar: 50 μm). Prostate (Prostate Bx), prostatectomy, transurethral resection of the prostate (TURP), lymph node (LN), and bone marrow trephine (BMT) biopsies are shown. (**B**) Nuclear AR-V7 expression (H score [HS]) in 63 same-patient matched CSPC (gray) and CRPC (red) biopsies from the ICR/RMH cohort. Three AR-null CRPC cases with neuroendocrine features are shown (blue). Median HS and interquartile range are shown. *P* value was calculated using Wilcoxon signed-rank test. (**C**) Nuclear AR-V7 expression (HS) in 295 prostatectomy samples before any AR-targeted therapy. Median HS and interquartile range are shown. (**D**) Nuclear AR-V7 expression (HS) in 160 CRPC biopsies (red) and dichotomized (orange) by before (40 biopsies) and after (120 biopsies) AA or E treatment. Three AR-null CRPC cases with neuroendocrine features are shown (blue). Twenty biopsies taken after progression on primary ADT (with or without bicalutamide) and prior to standard therapy for CRPC are shown (green). Median HS and interquartile range are shown. *P* value was calculated using Mann-Whitney test. (**E**) Nuclear AR-V7 expression (HS) in 160 CRPC biopsies (red) from lymph node (LN), bone (BMT), liver, prostate, and other sites of metastases. Three AR-null CRPC cases with neuroendocrine features are shown (blue). Median HS and interquartile range are shown. *P* value was calculated using nonparametric equality-of-medians test.

A recombinant rabbit monoclonal antibody (Clone RM7) was developed, in collaboration with RevMAb Biosciences, against CE3 of AR-V7. Antibody validation was performed at The Institute of Cancer Research/Royal Marsden Hospital (ICR/RMH) and the University of Washington (UW). Western blot analysis of AR-V7–positive cell lines (LNCaP95, 22Rv1, and VCaP) demonstrated a strong AR-V7 band at 80 kDa (Figure 1A and Supplemental Figure 1A; supplemental material available online with this article; https://doi.org/10.1172/JCI122819DS1). In contrast, no band was seen in AR-V7–negative cell lines (LNCaP, PC3, and DU145) at 80 kDa (Figure 1A and Supplemental Figure 1A). We next compared RM7 with EPR15656, an AR-V7 antibody that has been studied in PC tissue and circulating tumor cells (CTCs), and used for biomarker studies of treatment stratification in CRPC (27, 28, 30). EPR15656 primarily recognizes AR-V7 but may also bind other proteins demonstrating staining in PC3 cells, colorectal (liver) metastasis, normal lung epithelium, and cytoplasmic compartment (2, 28, 30). EPR15656 demonstrated a strong AR-V7 band at 80 kDa in LNCaP95, 22Rv1, and VCaP (Figure 1A and Supplemental Figure 1A). However, consistent with reports of positive staining in PC3 cells, EPR15656 demonstrated a strong band at 70 kDa in PC3 (30). Following initial validation, specificity and increased affinity (compared with EPR15656) of RM7 for AR-V7 was confirmed by immunoprecipitation using M12–cumate-inducible AR-V7 cells demonstrating a single band at 80 kDa (Figure 1B). RM7 detected additional bands at approximately 150 kDa and 32 kDa. However, both the strong 80-kDa AR-V7 band and the faint 32-kDa band disappeared upon shRNA induction by doxycycline, suggesting that the 32-kDa band is a degradation product of AR-V7 (Figure 1C). In addition, the 150-kDa band was not seen by Western blot analysis using an alternative extraction method at UW (Figure 1C). Furthermore, when RM7 was optimized for immunohistochemistry (IHC), AR-V7–expressing cell lines (22Rv1, LNCaP95, and VCaP) were positive for AR-V7 by IHC and AR-V7–negative cell lines (LNCaP, DU145, and PC3) were negative for AR-V7, confirming that the 150-kDa band (present in LNCaP) was not recognized by IHC and RM7 does not stain PC3 cells (Figure 1D). In addition, RM7 stained neither a colorectal cancer (liver) metastasis nor normal lung epithelium, which stained positive with EPR15656 previously (Supplemental Figure 1B and ref. 30). Having confirmed that RM7 recognizes AR-V7, we performed IHC on formalin-fixed, paraffin-embedded PC patient tissue biopsies within our study cohorts, demonstrating strong, almost exclusively, nuclear staining (Figure 2 and Figure 3A). In contrast, EPR15656 demonstrated cytoplasmic staining in PC tissue and CTCs (27, 28, 30). Taken together, these data demonstrate that RM7 specifically recognizes AR-V7 protein in tissue biopsies from PC patients, with reduced off-target liabilities compared with EPR15656.

### Primary prostate cancers rarely express AR-V7 protein.

Previous studies have demonstrated AR-V7 mRNA and protein expression in primary PC (36–39). Having validated and optimized RM7 for IHC on PC patient samples, we investigated nuclear AR-V7 protein expression in early PC specimens. We used the ICR/RMH and UW CSPC cohorts (Figure 2). A single biopsy (1.6%) of 63 CSPC biopsies (ICR/RMH CSPC cohort) expressed nuclear AR-V7 (Figure 3B and Supplemental Table 1). Similarly, in 295 primary PC specimens (UW CSPC cohort) from men who were treated with a radical prostatectomy, and had not received AR-directed therapy, there were no (0%) nuclear AR-V7–positive cases (Figure 3C). Clinical data (for prostate-specific antigen progression–free survival) were available on 128 patients from the UW CSPC cohort (Supplemental Table 2). Of the 128 patients with 5-year follow-up, 64 had biochemical recurrence; none of these 64 patients had detectable AR-V7 protein in their initial prostatectomy tissue. These data confirm that AR-V7 is rarely (0.3%; 1 of 358) expressed and therefore nuclear AR-V7 protein expression cannot predict disease recurrence in radically treated primary PC.

### AR-V7 protein emerges as PC patients and mouse xenografts progress to castration-resistant disease and develop resistance to AA or E therapy.

Having demonstrated that nuclear AR-V7 protein is infrequently expressed in CSPC, we next explored nuclear AR-V7 expression in same-patient, matched biopsies, as 63 patients progressed from CSPC to CRPC (Figure 2). Nuclear AR-V7 protein significantly (*P* < 0.001) increased from CSPC (median H score, interquartile range [IQR]: 0, 0–0) to CRPC (H score, IQR: 70, 5–130) (Figure 3B). Next, we expanded this cohort to 160 CRPC biopsies (Figure 2). Median nuclear AR-V7 expression was (H score) 75, (IQR) 5–130 (Figure 3D and Supplemental Table 3); three (1.9%) metastatic CRPC (mCRPC) biopsies of 160 cases had neuroendocrine-like features and were negative for both nuclear AR-FL and nuclear AR-V7 expression. We next determined whether nuclear AR-V7 expression altered as patients progressed through standard-of-care AR-targeting therapies for CRPC (Figure 3D). Interestingly, 15 (75%) of 20 biopsies taken after progression on primary ADT (with or without bicalutamide) before the starting of standard systemic therapy for CRPC had detectable nuclear AR-V7 protein expression (H score, IQR: 40, 1.25–92.5) (Figure 3D). Furthermore, nuclear AR-V7 expression was significantly lower (*P* = 0.020) in 40 biopsies prior to AA or E therapy (H score, IQR: 40, 1–107.5) than in 120 biopsies after AA or E therapy (H score, IQR: 90, 20–150) (Figure 3D). There was no clear association between nuclear AR-V7 expression and time of biopsy after starting of AA or E therapy (*r* = –0.11 [–0.31 to 0.09], *P* = 0.27) (Supplemental Figure 2). Next we determined whether nuclear AR-V7 expression differed between sites of CRPC biopsy. Nuclear AR-V7 expression was higher (*P* = 0.013) in lymph node metastases (H score, IQR: 120, 60–170) compared with bone (H score, IQR: 50, 1–110), liver (H score, IQR: 70, 3.75–132.5), prostate (H score, IQR: 50, 0–70), and other (H score, IQR: 90, 0–150) sites of metastases (Figure 3E). Finally, we investigated whether the same pattern of nuclear AR-V7 expression was observed as VCaP (androgen-dependent) mouse xenograft models developed therapeutic resistance. Consistent with our tissue studies, nuclear AR-V7 expression increased in VCaP mouse xenografts as they progressed from the castration-sensitive (H score, IQR: 0, 0–0) to the castration-resistant state (H score, IQR: 155, 102.5–175) to AA/E resistance (H score, IQR: 180, 160–190) (Supplemental Figures 3 and 4). Although nuclear AR-V7 protein is rarely expressed in primary PC, AR-V7 protein expression emerges as patients and mouse xenografts progress to castration-resistant disease, and levels increase further as resistance to AA or E therapy develops.

### AR-V7 protein expression associates with response to AA and E, but not docetaxel, in CRPC.

**Figure 4:**
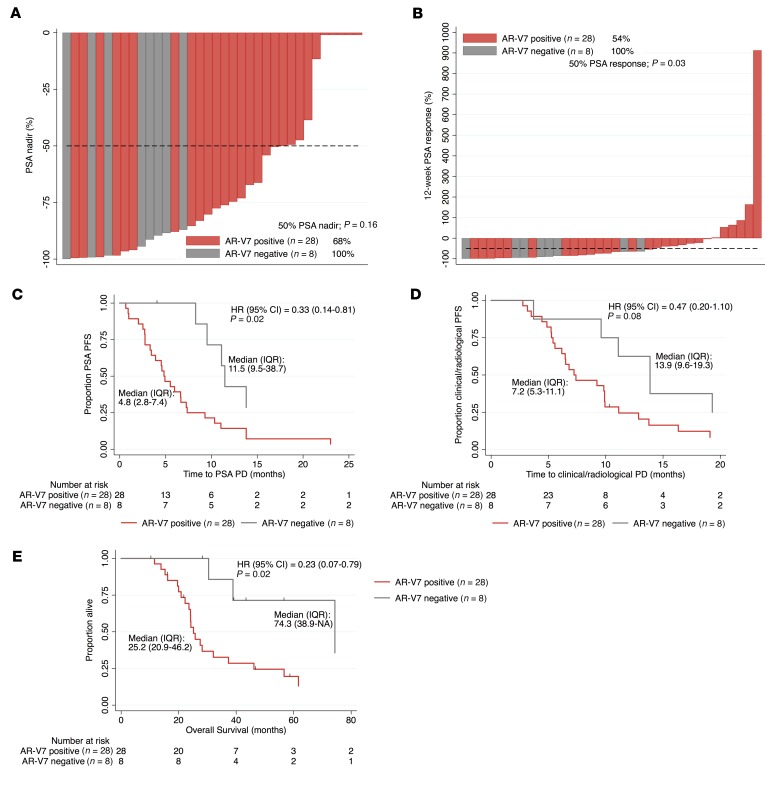
AR-V7 status and response to AR-targeting therapies (AA or E) prior to chemotherapy in CRPC. Thirty-six patients received AR-targeting therapies (AA or E) prior to chemotherapy for CRPC. (**A**) Percentage PSA nadir on AR-targeting therapies for AR-V7–negative (H score ≤ 10; gray) and AR-V7–positive (H score > 10; red) CRPC patients is shown. Fifty percent PSA nadir rate is shown. *P* value was calculated using Fisher’s exact test. (**B**) Percentage 12-week 50% PSA response rate on AR-targeting therapies for AR-V7–negative (gray) and AR-V7–positive (red) CRPC patients is shown. Twelve-week 50% PSA response rate is shown. *P* value was calculated using Fisher’s exact test. (**C**–**E**) Kaplan-Meier curves show time to PSA progression (**C**), time to clinical/radiological progression (**D**), and overall survival (**E**) from start of AR-targeting therapy. Hazard ratios (HRs) with 95% confidence intervals (CIs) are shown. *P* value was calculated using univariate Cox proportional hazards model.

Studies have shown AR-V7 protein and mRNA to be a marker of next-generation AR-targeted therapy (AA and E) resistance (19, 20, 26–30). To investigate this further, we determined the response of the ICR/RMH CRPC cohort to AR-targeted therapies before and after chemotherapy in AR-V7–negative (nuclear H score ≤ 10) and AR-V7–positive (nuclear H score > 10) patients. Thirty-six patients received AA or E for CRPC prior to chemotherapy and had fully evaluable response data (Figure 2). Patients negative for AR-V7 expression were younger (*P* = 0.04) at the time of starting AR-targeting therapy before chemotherapy, but no other differences in baseline characteristics were observed (Table 1). Patients negative for AR-V7 (*n* = 8) had a greater prostate-specific antigen (PSA) 50% nadir (100% vs. 68%, *P* = 0.16) and PSA 50% response rate (100% vs. 54%, *P* = 0.03) than AR-V7–positive patients (*n* = 28) (Figure 4, A and B). Patients achieving a 50% PSA fall had significantly lower (*P* = 0.012) nuclear AR-V7 expression (H score, IQR: 40, 0–100) than those who did not (H score, IQR: 120, 55–180) (Supplemental Figure 5). Furthermore, AR-V7–negative patients had a longer time to PSA progression (11.5 vs. 4.8 months, hazard ratio [HR] 0.33 [0.14–0.81], *P* = 0.02), longer time to clinical/radiological progression (13.9 vs. 7.2 months, HR 0.47 [0.20–1.10], *P* = 0.08), and improved overall survival (74.3 vs. 25.2 months, HR 0.23 [0.07–0.79], *P* = 0.02) (Figure 4, C–E). Fifty-four patients received AA or E for CRPC after chemotherapy and had fully evaluable response data (Figure 2). There were no differences in the baseline characteristics by AR-V7 status at the time of starting AR-targeting therapy after chemotherapy (Supplemental Table 4). Patients negative for AR-V7 (*n* = 17) had a significantly greater PSA 50% nadir (71% vs. 24%, *P* = 0.002) and PSA 50% response rate (59% vs. 22%, *P* = 0.012) than those positive for AR-V7 (*n* = 37) (Supplemental Figure 6, A and B). Patients achieving a 50% PSA fall had significantly lower (*P* = 0.011) nuclear AR-V7 expression (H score, IQR: 3, 0–80) than those who did not (H score, IQR: 95, 25–125) (Supplemental Figure 6C). Interestingly, despite these significant differences in response rates, there was no significant difference in time to PSA progression (2.8 vs. 2.3 months, HR 0.96 [0.54–1.73], *P* = 0.90), time to clinical/radiological progression (4.9 vs. 5.1 months, HR 0.92 [0.51–1.66], *P* = 0.77), or overall survival (14.0 vs. 15.7 months, HR 1.01 [0.56–1.82], *P* = 0.98) (Supplemental Figure 6, D–F). Having explored response to AR-targeted therapy, we next investigated response to docetaxel chemotherapy. Fifty-five patients were treated with docetaxel chemotherapy for CRPC and had fully evaluable response data (Figure 2). There was no evidence of a difference in baseline characteristics at the time of starting docetaxel chemotherapy (Supplemental Table 5). In contrast to AR-targeting therapies, there was no difference in PSA 50% nadir (56% vs. 46%, *P* = 0.57) and PSA 50% response rate (39% vs. 27%, *P* = 0.54) between AR-V7–negative (*n* = 18) and –positive patients (*n* = 37) (Supplemental Figure 7, A and B). Nuclear AR-V7 expression was not significantly (*P* = 0.14) different in patients achieving a 50% PSA fall with docetaxel (H score, IQR: 20, 0–85) compared with those who did not (H score, IQR: 70, 5–132.5) (Supplemental Figure 7C). Consistent with this, there was no significant difference in time to PSA progression (4.8 vs. 4.7 months, HR 1.04 [0.57–1.92], *P* = 0.90) and time to clinical/radiological progression (6.9 vs. 7.5 months, HR 1.31 [0.73–2.34], *P* = 0.36) (Supplemental Figure 7, D and E). However, AR-V7–negative patients had improved overall survival (26.3 vs. 18.5 months, HR 0.50 [0.27–0.95], *P* = 0.03) compared with AR-V7–positive patients (Supplemental Figure 7F). Taken together, these data confirm that AR-V7 is a robust prognostic biomarker and an important indicator of sensitivity to AR-targeted therapies but not docetaxel treatment in CRPC.

### AR-FL and AR-V7 mRNA and protein expression associate in a high proportion of, but not all, CRPCs.

**Figure 5:**
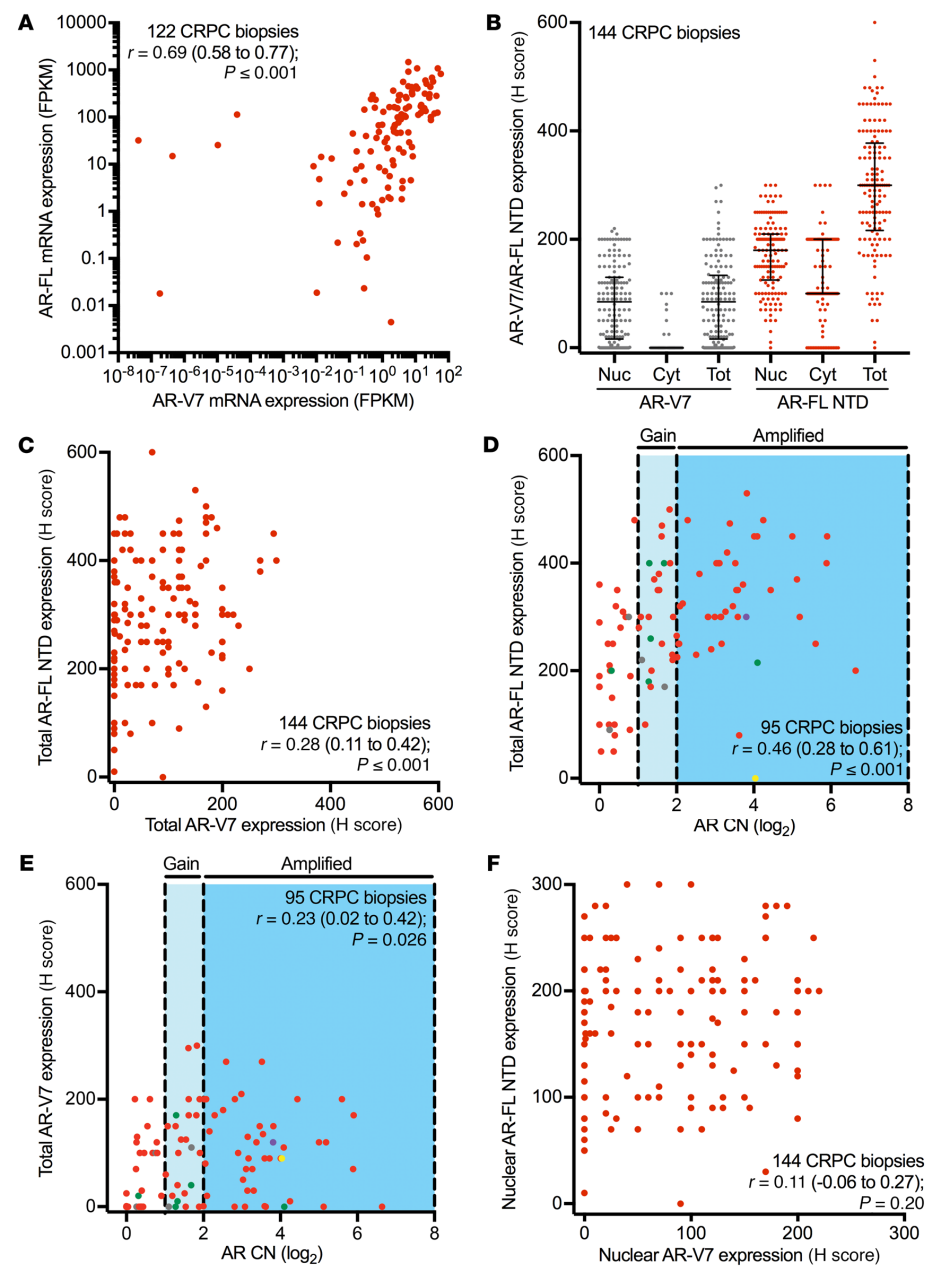
AR-FL and AR-V7 mRNA and protein quantification with AR copy number analysis in CRPC. (**A**) AR-FL and AR-V7 mRNA expression in fragments per kilobase of transcript per million mapped reads (FPKM) for 122 CRPC transcriptomes from the SU2C/PCF cohort is shown. Spearman’s rank correlation is shown. (**B**) Expression (H score [HS]) for nuclear, cytoplasmic, and total (nuclear + cytoplasmic) AR-V7 (gray) and AR-FL N-terminal domain (NTD; red) is shown. Median HS and interquartile range are shown. (**C**) Expression (HS) of total AR-FL NTD protein and total AR-V7 protein in 144 CRPC biopsies from the ICR/RMH CRPC cohort is shown. Spearman’s rank correlation is shown. (**D**) Expression (HS) of total AR-FL NTD protein and AR copy number (log_2_) in 95 CRPC biopsies from the ICR/RMH CRPC cohort are shown. Cases with AR mutations are shown (L702H gray, T878A green, H875Y purple, K313E yellow). Spearman’s rank correlation is shown. (**E**) Expression (HS) of total AR-V7 protein and AR copy number (log_2_) in 95 CRPC biopsies from the ICR/RMH CRPC cohort are shown. Cases with AR mutations are shown. Spearman’s rank correlation is shown. (**F**) Expression (HS) of nuclear AR-FL NTD protein and nuclear AR-V7 protein in 144 CRPC biopsies from the ICR/RMH CRPC cohort is shown. Spearman’s rank correlation is shown.

We and others have shown that AR-FL and AR-V7 mRNA and protein are induced upon castration, and that therapies suppressing mRNA splicing prevent AR-V7 mRNA and protein generation in CRPC (33, 44–47). Therefore, we next investigated the association between AR-FL and AR-V7 mRNA and protein expression (Figure 2). Analysis of RNA sequencing (RNA-Seq) data obtained from 122 CRPC biopsies demonstrated that AR-FL mRNA expression significantly correlated with AR-V7 mRNA expression (*r* = 0.69 [0.58–0.77], *P* ≤ 0.001) (Figure 5A). In light of RNA quantification not discriminating against cellular localization, we next quantified total (nuclear and cytoplasmic) AR-FL and AR-V7 protein expression in 144 CRPC biopsies (ICR/RMH CRPC cohort) (Figure 5B and Supplemental Figure 8). Unlike AR-FL, of which 124 of 144 (86%) biopsies had both cytoplasmic and nuclear AR-FL protein expression, AR-V7 protein was almost exclusively (136/144; 94% of biopsies) nuclear in localization (Figure 5B). There was a significant correlation between total AR-FL and AR-V7 protein expression in 144 CRPC biopsies from the ICR/RMH CRPC cohort (*r* = 0.28 [0.11–0.42], *P* ≤ 0.001) (Figure 5C). However, it is important to recognize that a substantial number of patients with high AR-FL mRNA and protein expression had low or undetectable levels of AR-V7 mRNA and protein. Furthermore, both total AR-FL (*r* = 0.46 [0.28–0.61], *P* ≤ 0.001) and total AR-V7 (*r* = 0.23 [0.02–0.42], *P* = 0.026) protein significantly correlated with AR copy number in 95 CRPC biopsies from the ICR/RMH CRPC cohort (Figure 5, D and E). Finally, consistent with the demonstration that AR-FL and AR-V7 are differentially localized, there was no significant correlation between nuclear AR-FL and nuclear AR-V7 protein expression in 144 CRPC biopsies from the ICR/RMH CRPC cohort (*r* = 0.11 [–0.06 to 0.27], *P* = 0.20) (Figure 5F). These data demonstrate that, taken together, total AR-FL and AR-V7 mRNA and protein expression correlate in CRPC biopsies, although, importantly, many patients have tumors expressing high levels of AR-FL mRNA and protein but that have low or undetectable AR-V7 mRNA and protein expression. This suggests that the presence of AR-V7 mRNA and protein is not simply a consequence of higher AR-FL levels in all cases.

### AR-V7 protein expression is largely homogenous within metastasis but heterogeneous between metastases from patients with CRPC.

**Figure 6:**
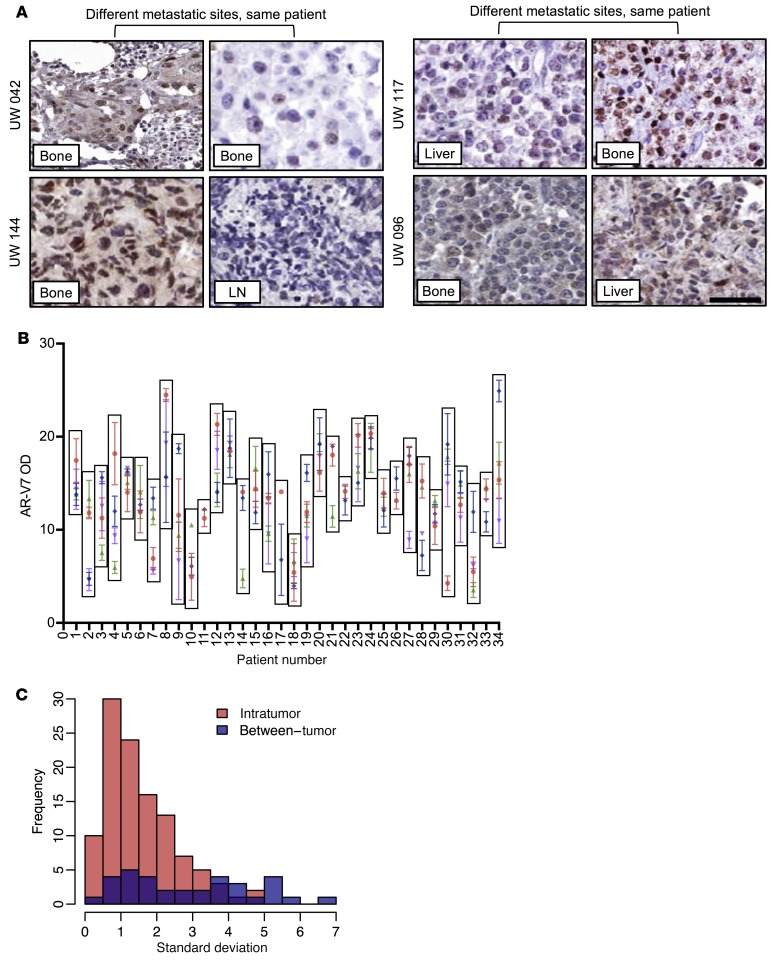
AR-V7 protein expression variability within metastasis and between metastases from individual patients with CRPC. (**A**) Representative micrographs of AR-V7 detection by IHC in 4 UW patients with multiple CRPC biopsies (original magnification, ×200; scale bar: 50 μm). (**B**) Nuclear AR-V7 expression (OD) in 133 metastases from 34 CRPC patients from the UW CRPC cohort. Mean OD and standard deviation (SD) for 3 measurements from each metastasis are shown. Each box encloses all metastases from a patient. Different colors for each patient represent an individual metastasis. (**C**) Frequency distribution of SD within a metastasis (Intratumor; comparison of triplicates in a metastasis; red) and between metastases (Between-tumor; comparison of multiple metastasis within a patient; blue) is shown. Median SD is 1.2 for intratumor measurements and 2.9 for between-tumor measurements.

We have previously shown that in a patient with a genomic rearrangement resulting in the constitutively active AR^v567es^ variant, each of 5 metastases expressed the variant AR in a homogenous fashion (48). Since AR-V7 is usually generated not from a structural rearrangement of the AR gene but rather from aberrant mRNA splicing, we next quantitated expression in 133 metastases from 34 CRPC patients that were collected as part of the University of Washington Medical Center Prostate Cancer Donor Rapid Autopsy Program (UW CRPC cohort) (Figure 2, Figure 6A, and Supplemental Table 6). Automated digital scoring reported as optical density (OD) correlated significantly (*r* = 0.86 [0.83–0.89], *P* ≤ 0.001) with manual scoring and was used to determine nuclear AR-V7 expression in the UW CRPC cohort (Supplemental Figure 9, A–C). Three tissue microarray spots were stained from each metastasis and AR-V7 levels quantified. We found that expression of AR-V7 was largely consistent within each metastasis from a patient and that the variance was not statistically significant (Fligner-Killeen *P* = 0.9999, Levene’s *P* = 0.9972) (Figure 6, B and C). However, expression of AR-V7 in different metastases in an individual patient differed widely, and the variance was statistically significant (Fligner-Killeen *P* = 3.73 × 10^–06^, Levene’s *P* = 3.25 × 10^–07^) (Figure 6, B and C). These data suggest that within an individual patient the degree to which AR-V7 may be driving different metastases varies and may result in mixed response to endocrine therapies.

### AR-V7 expression is associated with a unique gene signature in CRPC.

**Figure 7:**
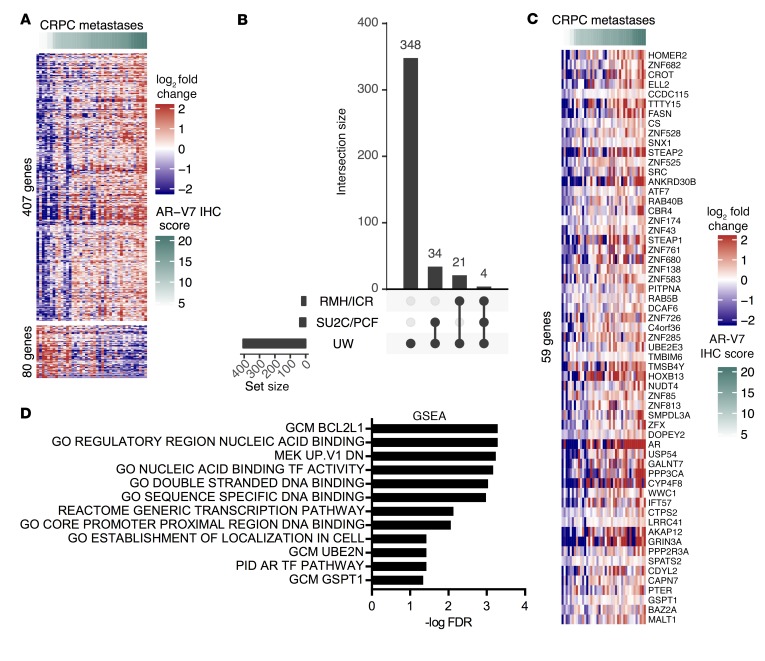
AR-V7 protein expression is associated with a unique gene signature in metastatic CRPC. (**A**) Expression (OD) of nuclear AR-V7 protein correlated (*q* < 0.05) with gene mRNA expression of 487 (407 upregulated and 80 downregulated) genes in 41 metastases from 24 patients from the UW CRPC cohort. Heatmap shows metastases ranked in order of nuclear AR-V7 expression (OD) and mean-centered log_2_ fold change in gene mRNA expression. (**B**) Fifty-nine of the 407 upregulated genes were validated in either 21 CRPC metastases from the ICR/RMH CRPC cohort or 122 CRPC transcriptomes from the SU2C/PCF cohort. Figure shows overlap of significantly correlated genes between the 3 cohorts. (**C**) Heatmap shows metastases ranked in order of nuclear AR-V7 expression (OD) and mean-centered log_2_ fold change in gene mRNA expression of the 59-gene signature in the UW CRPC cohort (*n* = 41). (**D**) Pathway overrepresentation analysis using MSigDB v6.2 (H, Hallmark; CP, Canonical Pathways; C4, Computational Gene Sets; C5, GO; and C6, Oncogenic Pathway) in the 59-gene set. Pathways with FDR less than 0.05 are shown. GSEA, gene set enrichment analysis.

Having demonstrated interpatient and intrapatient heterogeneity in nuclear AR-V7 expression, we next investigated whether nuclear AR-V7 expression was associated with a specific gene signature in CRPC patients. Forty-one metastatic biopsies from 24 men within the UW CRPC cohort had mRNA expression (RNA-Seq) and AR-V7 protein expression (IHC) available. The correlation between AR-V7 protein expression (OD) and gene mRNA expression (log_2_ counts per million) was determined and corrected for multiple testing. We identified 487 genes that correlated (*q* < 0.05) with AR-V7 protein expression; of these, 407 positively correlated and 80 negatively correlated (Figure 7A). Pathway analysis of the 407 genes that positively correlated with AR-V7 protein expression identified an enrichment for pathways involved in transcription and the androgen response (Supplemental Figure 10, Supplemental Table 7, and refs. 49, 50). We confirmed that AR-V7 mRNA expression and AR-V7 protein expression correlated significantly in 41 UW (*P* < 0.001) and 21 ICR/RMH (*P* = 0.004) CRPC biopsies (data not shown). Next we independently tested the positively correlated 407-gene signature in 21 CRPC metastases from an ICR/RMH CRPC cohort and 122 CRPC tumor transcriptomes (International Stand Up To Cancer/Prostate Cancer Foundation [SU2C/PCF] cohort) (Supplemental Table 8 and ref. 51). Of the genes identified, 59 were found to be significantly correlated with nuclear AR-V7 protein expression in the ICR/RMH cohort or with AR-V7 mRNA expression in the SU2C/PCF cohort (Figure 7, B and C, and Supplemental Table 9). Following this, pathway analysis of the 59 independently validated genes confirmed a role in transcriptional activity (Figure 7D and Supplemental Table 10). Consistent with this finding, 33% were zinc finger–containing (ZNF) genes correlated with chromatin binding, including HOXB13, ELL2, STEAP2, and BAZ2A. Furthermore, a large number of the genes identified have been associated with PC progression (Supplemental Table 9 and refs. 52–64). In addition, genome-wide analyses demonstrated that AR-V7 protein expression associates with AR gene expression (Figure 7C). Although an association between AR-FL mRNA and AR-V7 mRNA using junction-specific reads was confirmed, there was further confirmation that a substantial number of cases that express high AR-FL mRNA levels have low or undetectable AR-V7 mRNA (Supplemental Figure 11). These data suggest that AR-V7 expression is associated with a specific gene signature in a large patient population that may play a key role in transcriptional activity and PC progression in patients with CRPC.

## Discussion

Since the pioneering studies of Huggins and Hodges in 1941, the androgen receptor has remained the focus of therapeutic targeting in CRPC. Inhibition of the AR axis with AA and E has improved both overall survival and quality of life for patients with CRPC (10–17, 65). Although these modalities are initially effective, resistance develops with ongoing AR activity and tumor progression. This is, at least in part, due to the expression of constitutively active AR splice variants, of which AR-V7 appears to be the most common (19–32). AR-V7 mRNA and protein expression has been detected at low levels in primary, treatment-naive PC, and studies have reported a potential association with worse outcome (36–39). Surprisingly, in 2 separate patient cohorts we found that nuclear AR-V7 protein was expressed in fewer than 1% of PC tumors at diagnosis, and therefore it cannot be predictive of outcome after primary therapy. The difference in prevalence of AR-V7 protein expression reported in different studies is likely attributable to the different AR-V7 antibodies used, and in particular, the observation that one antibody used has off-target liabilities as we have previously reported (30). Consistent with this, we demonstrate AR-V7 protein expression to be almost exclusively nuclear, whereas previous studies have demonstrated cytoplasmic positivity (27, 28, 30). These data suggest that AR-V7 testing is unlikely to be of use for treatment stratification at time of diagnosis and may be better utilized beyond first-line treatment (11, 18).

In contrast to primary PC, 75% of CRPC patients who had progressed on primary ADT alone (with or without bicalutamide) expressed nuclear AR-V7 before receiving AA or E. Despite AR-V7 expression after primary ADT, subsequent AA or E treatment has significant antitumor activity with response rates ranging from 57% to 78% (12, 14, 66). These data indicate that AR-V7 protein expression in biopsies cannot indicate absolute refractoriness to treatment. Although, consistent with previous reports, nuclear AR-V7 levels increased further in response to AA or E, these data suggest that AR-V7 expression is a factor at the initial phase of castration resistance following primary ADT in advanced PC (30, 40). These data were further confirmed in VCaP mouse xenograft models as they developed resistance to castration and AA/E therapy. Recent studies have shown AA therapy at diagnosis to improve overall survival in PC patients with de novo metastatic disease (11, 18). The demonstration that nuclear AR-V7 expression is rare in primary PC, but emerges with primary therapy, may provide insight into the greater efficacy of AA in CSPC. Importantly, these data show that increased AR-V7 expression is an early event in resistance, and if agents targeted to AR splice variants become clinically available, therapy may need to be combined at the time of initial ADT.

Critically, we found that AR-V7 protein expression is more prevalent in CRPC biopsies than previously reported from AR-V7 mRNA and protein expression studies in liquid biopsies (11%–46%) (19, 20, 27–29). This important observation is likely due to the differences in sensitivities of the assays used. In addition, CRPC biopsies demonstrate lymph node metastases to express higher levels of AR-V7 than other sites of disease, which may (depending on the source of CTCs) account for the lower incidence of AR-V7 detected in liquid biopsies. Furthermore, we demonstrate intrapatient heterogeneity of nuclear AR-V7 expression between multiple metastases, indicating that this is a further potential source of variation in CTC-based AR-V7 assessment. Finally, depending on the biomarkers used to select CTCs, assessment of AR-V7 may be underestimated if not all CTCs are identified. These findings suggest that the detection of AR-V7 in CTCs may not be representative of all metastases, and that while sites of disease expressing AR-V7 may be resistant to current endocrine therapies, those expressing low AR-V7 or no AR-V7 may still respond, within the same subject. Additional studies focusing on the prognostic value of tissue-based AR-V7 detection in CTC-negative patients may be warranted.

The majority of clinical studies have demonstrated that AR-V7 positivity confers resistance and poorer outcome to AR-targeting therapies in patients with CRPC (19, 20, 26–31). We confirmed that AR-V7–positive patients treated with AA or E had a worse PSA response rate in the pre- and post-chemotherapy setting; and those patients who responded had lower levels of AR-V7 expression. Interestingly, despite poorer response rates, only AR-V7–positive patients treated with AA or E before chemotherapy had shorter progression-free and overall survival. This observation could be multifactorial. Firstly, not all patients had tissue biopsies before starting treatment, and therefore AR-V7 status may have changed before therapy. Secondly, patients without CTCs and therefore no AR-V7 result may be underrepresented in previous studies. In contrast to endocrine therapies, AR-V7 status did not associate with PSA response or progression-free survival in patients treated with docetaxel, as previously described (27, 28, 67). Interestingly, AR-V7–positive patients had shorter overall survival, suggesting that AR-V7 positivity may be associated with more aggressive disease, or this group of patients may have derived less benefit from treatment with further novel endocrine therapies. Taken together, these studies will be important to understand, as the landscape of CRPC changes as patients with de novo metastatic CSPC receive AA, and as we explore the potential use of AR-V7 to stratify patients to further AR-targeted therapies as they progress to CRPC.

The mechanisms by which AR splice variants are generated include genomic rearrangements and/or aberrant alternative mRNA splicing (22, 24, 32, 48, 68–71). AR-V7 generation has generally been attributed to aberrant splicing of AR pre-mRNA (72). This does not negate the fact that AR-FL increases under conditions such as castration, and that this leads to the generation of AR-V7 (33, 46, 47). However, these data indicate that the mechanisms driving increased AR-FL expression and AR signaling in CRPC differ from those required for AR-V7 generation (72–76). Consistent with this, we demonstrate that although AR-FL and AR-V7 expression associate in CRPC, a substantial number of patients with high levels of AR-FL demonstrate undetectable or low levels of AR-V7 expression. In keeping with this also is evidence emerging that therapies that suppress mRNA splicing decrease AR-V7 generation in CRPC models (33, 45). JMJD1A has recently been reported to be critical for mRNA splicing and AR-V7 generation but does not impact on AR-FL levels (77). These data suggest that mRNA splicing is important for AR-V7 generation and is not simply a consequence of increased AR-FL expression. Further understanding of the mechanisms underpinning AR-V7 generation are now required to support the development of therapeutic strategies to suppress splice variant generation in CRPC.

Previous studies examining AR-V7 cistromes and transcriptomes demonstrated proliferative cistomes/transcriptomes that are likely not specific to AR-V7 but a marker of rapidly progressive disease (23, 78–80). Although a recent study suggested that CRPC transcriptomes are diverse, we identified 59 genes using 3 independent patient cohorts that associate with AR-V7 expression (79). One important consideration is that AR-V7 protein expression associates with AR gene expression in genome-wide analysis. However, as both AR-V7 and AR-FL mRNA would be represented in such studies, further junction-specific quantification was performed. Despite this approach confirming a correlation between AR-FL and AR-V7 mRNA, there was further evidence of dissociation in many cases. In addition, a gene signature was derived from AR-V7 protein expression; unlike AR-FL protein, AR-V7 is almost exclusively nuclear, and therefore, unlike mRNA analysis, this takes into consideration its likely functional importance. Interestingly, BAZ2A, SRC, STEAP1, STEAP2, DCAF6, TMBIM6, HOXB13, GALANT7, WWC1, SPATS2, and GSTP1 expression associated with AR-V7 expression, and all of these have been previously linked to PC progression (52–64). In addition, HOXB13 has recently been shown to be critical for AR-V7 chromatin binding (79). Furthermore, we found a number of ZNF contigs to be associated with AR-V7 expression, providing evidence of increased transcriptional activity. These data suggest that AR-V7 protein expression is associated with a unique gene signature important for PC progression and transcriptional activity. It is important to stress that the 59-gene set derived does not represent the AR-V7 cistrome but is a set of genes associated with AR-V7 expression between cohorts and may identify common characteristics of AR-V7–associated disease. Components of this gene signature, including HOXB13, which has recently been shown to be critical for AR-V7 function, may provide insight into therapeutic targets for novel treatment strategies in patients with high levels of AR-V7 expression (79).

In conclusion, our results show that AR-V7 protein expression, using a validated, highly specific antibody, is not seen in primary CSPC and does not appear until initial resistance to standard ADT occurs, and increases further with AA and E therapy. In addition, AR-V7 protein expression associates with resistance to AR-targeted therapies but not taxane treatment in patients with CRPC. Furthermore, although AR-V7 and AR-FL expression levels associate in CRPC, there are many cases in which expression levels are uncoupled, suggesting that AR-V7 protein expression is not simply a function of AR-FL protein expression. Moreover, AR-V7 protein is heterogeneously expressed, especially between metastases from the same patient, indicating multiple resistance mechanisms in the same subject. These data suggest that multiple therapeutic modalities may be needed simultaneously to adequately reverse endocrine resistance in AR-V7–positive PC. Finally, AR-V7 protein expression associates with a unique gene signature that may drive transcriptional activity and PC progression. These results further confirm the importance of AR-V7 in CRPC biology and provide impetus for the development of novel therapeutic strategies that abrogate AR-V7 expression at the time of initial ADT in CSPC in order to prevent or delay development of CRPC and improve the outcome for patients with lethal PC.

## Methods

### Cell lines

LNCaP95 cells were provided by Alan K. Meeker and Jun Luo (Johns Hopkins University, Baltimore, Maryland, USA). 22Rv1 (CRL-2505), VCaP (CRL-2876), DU145 (HTB-81), M12 (a gift from Joy Ware, Virginia Commonwealth University, Richmond, Virginia, USA), and PC3 (CRL-1345) cells were obtained from American Type Culture Collection. Doxycycline-inducible cell lines were created using lentiviral vectors in pLKO-Tet-On backbones targeting either GFP (shGFP; 5′-GCAAGCTGACCCTGAAGTTCA-3′), AR-FL exon 8 (shAR-FL; 5′-CCTGCTAATCAAGTCACACAT-3′), or AR-V7 cryptic exon 3 (shAR-V7; 5′-GTAGTTGTGAGTATCATGA-3′), and lentiviral particles were produced as previously described (81, 82). Cells were infected with virus and selected with 1 μg/ml puromycin. shRNA expression was induced by treatment of cells with 1 μg/ml doxycycline for 72 hours. All cell lines were grown in recommended media at 37°C in 5% CO_2_. Cell lines were tested for mycoplasma using the VenorGem One Step PCR Kit (Cambio) and short tandem repeat (STR) profiled.

### Immunoblotting

#### ICR/RMH antibody validation.

Cells were lysed with RIPA buffer (Pierce) supplemented with protease inhibitor cocktail (Roche). Protein extracts (20 μg) were separated on 7% NuPAGE Tris-Acetate gel (Invitrogen) by electrophoresis and subsequently transferred onto Immobilon-P PVDF membranes of 0.45 μm pore size (Millipore). Primary antibodies used were rabbit monoclonal anti–AR-V7 (1 in 1,000; RM7, RevMAb Biosciences), rabbit monoclonal anti–AR-V7 (1 in 1,000; EPR15656, Abcam), and mouse monoclonal anti-vinculin (1 in 200,000; V9131, Sigma-Aldrich) with species-specific secondary antibodies conjugated to horseradish peroxidase. Chemiluminescence was detected on the Chemidoc Touch imaging system (Bio-Rad).

#### UW antibody validation.

Cells were lysed with M-PER Mammalian Protein Extraction Reagent (Thermo Fisher Scientific) supplemented with Halt Protease Inhibitor and Halt Phosphatase Inhibitor Cocktail. Protein extracts (30 μg) were separated on 4%–15% Mini-PROTEAN TGX Precast Protein Gel (Bio-Rad) by electrophoresis and subsequently transferred to a nitrocellulose membrane with an iBlot system. Primary antibodies used were rabbit monoclonal anti–AR-V7 (1 in 2,000; RM7, RevMAb Biosciences), mouse monoclonal anti–AR N-terminus (1 in 2,000; AR441, Santa Cruz Biotechnology), and rabbit monoclonal anti-GAPDH (1 in 10,000; 2118, Cell Signaling Technology). The specific signals were visualized on Blue Ultra Autorad Film (GeneMate) with species-specific secondary antibodies conjugated to horseradish peroxidase by chemiluminescence.

### AR-V7 immunoprecipitation

Cellular extracts were prepared from cumate-treated M12 cells expressing cumate-inducible AR-V7 lentivirus using the SparQcumate switch lentivector system (Systems Bioscience) as previously described (30). Precleared cell lysate was incubated with rabbit monoclonal anti–AR-V7 antibodies (EPR15656, Abcam; or RM7, RevMAb Biosciences). Rabbit IgGs were used as a negative control. Immune complexes were collected using protein A/G Plus agarose beads and analyzed by immunoblotting as described above.

### VCaP mouse xenograft models

All animal studies were performed in accordance with Beth Israel Deaconess Medical Center IACUC regulations (protocol 086-2016). VCaP mouse xenograft models have been previously described (83). Briefly, 5 million VCaP cells in 100% Matrigel were injected subcutaneously into 6-week-old ICR scid mice (Taconic Biosciences). Xenografts were grown until 1,000 mm^3^; then mice were castrated. For AA- and E-resistant xenograft model, when castrated tumors exceeded 150% nadir volume, they were treated with AA (30 mg/kg) and E (50 mg/kg). Tumors were biopsied before castration resistance, at castration resistance, and when resistant to AA and E therapy.

### ICR/RMH and UW tissue samples

The Institute of Cancer Research and Royal Marsden Hospital (ICR/RMH) CSPC and CRPC cohort was identified from men with CRPC treated at the Royal Marsden NHS Foundation Trust. The ICR/RMH CSPC cohort contained 63 patients with sufficient formalin-fixed, paraffin-embedded (FFPE) diagnostic (archival) CSPC biopsies; all biopsies demonstrated adenocarcinoma and were from either prostate needle biopsies (47 patients), transurethral resection of the prostate (TURP; 5 patients), transurethral resection of bladder tumor (TURBT; 1 patient), prostatectomy (8 patients), bone (1 patient), or rectal biopsy (1 patient). The ICR/RMH CRPC cohort contained 160 patients (which included all 63 patients in the CSPC cohort) with sufficient FFPE CRPC biopsies from metastatic biopsies of bone (81 patients), lymph node (51 patients), soft tissue (8 patients), liver (10 patients), TURP (7 patients), TURBT (1 patient), or prostate (2 patients). All tissue blocks were freshly sectioned and were only considered for IHC analyses if adequate material was present. Demographic and clinical data for each patient were retrospectively collected from the hospital electronic patient record system.

The University of Washington (UW) CSPC cohort was identified from men who received radical prostatectomy without neoadjuvant therapy. Tissue microarrays (TMAs) of FFPE tissue from primary prostate acinar adenocarcinomas were generated. The tissue came from the radical prostatectomy samples of 295 patients, none of whom had received neoadjuvant therapy. The TMAs consisted of single cores of 12 carcinomas, duplicate cores of 167 carcinomas, triplicate cores of 44 carcinomas, and quadruplicate cores of 72 carcinomas. The UW CRPC cohort was identified from men who died of their prostate cancer and were part of the University of Washington Medical Center Prostate Cancer Rapid Autopsy Program (5). The cohort consisted of a TMA generated from biopsies of 133 metastases from 34 patients. Triplicate cores of the 133 metastases were placed on the TMA.

### Immunohistochemistry

#### ICR/RMH CSPC and CRPC cohort.

AR-V7 and AR-FL IHC was performed as previously described (30, 31). Briefly, AR-V7 IHC was performed using recombinant rabbit monoclonal anti–AR-V7 antibody (Clone RM7, RevMAb Biosciences). Biopsies were first deparaffinized before antigen retrieval by microwaving (in Tris/EDTA buffer, pH 8.1) for 18 minutes at 800 W, and anti–AR-V7 antibody (1:500 dilution in Dako REAL diluent, Agilent Technologies) was incubated with tissue for 1 hour at room temperature. After washes, bound antibody was visualized using Dako EnVision Detection System (Agilent Technologies). Sections were counterstained with hematoxylin. Cell pellets from 22Rv1 (positive) and PC3 (negative) were used as controls. Rabbit IgGs were used as a further negative control. AR-FL IHC was performed using mouse monoclonal anti-AR antibody (AR441, Agilent Technologies). Biopsies were first deparaffinized before antigen retrieval using pH 8.1 Tris/EDTA solution heated in a water bath, and anti-AR antibody (1:1,000 dilution in Dako REAL diluent, Agilent Technologies) was incubated with tissue for 1 hour at room temperature. After washes, bound antibody was visualized using Dako EnVision Detection System (Agilent Technologies). Sections were counterstained with hematoxylin. Cell pellets from VCaP (positive) and PC3 (negative) were used as controls. Mouse IgGs were used as a further negative control.

#### UW CSPC and CRPC cohort.

Briefly, AR-V7 IHC was performed using recombinant rabbit monoclonal anti–AR-V7 antibody (Clone RM7, RevMAb Biosciences). Deparaffinization, antigen retrieval (Cell Conditioner 1, Ventana Medical Systems), and immunostaining were performed on the Ventana Benchmark automated stainer (Ventana Medical Systems). Sections were incubated for 2 hours at 37°C with anti–AR-V7 antibody (1:50 in antibody diluent; Ventana Medical Systems). After washes, bound antibody was visualized using Ventana Optiview DAB detection kit (Ventana Medical Systems). Sections were counterstained with hematoxylin. Controls were sections of a TMA made of cell lines known to express AR-FL and/or AR-V7 (LNCaP, 22Rv1, VCaP) or known not to express AR-FL or AR-V7 (DU145, PC3, M12) and cells engineered to stably express both AR-FL (M12 AR-FL) and AR-V7 (M12 AR-V7) by transfection.

### IHC quantification

#### ICR/RMH CSPC and CRPC cohort.

AR-V7 and AR-FL protein expression was determined for each case by a pathologist (D.N. Rodrigues) blinded to clinical data using the modified H score (HS) method, a semiquantitative assessment of staining intensity that reflects antigen concentration. HS was determined according to the formula: ([% of weak staining] × 1) + ([% of moderate staining] × 2) + ([% of strong staining] × 3), yielding a range from 0 to 300 (84).

#### UW CSPC and CRPC cohort.

AR-V7 protein expression in the UW CSPC cohort was determined for each case by a pathologist (L.D. True) as described above. AR-V7 protein expression in the UW CRPC cohort was determined using automated digital scoring as follows: TMA slides were scanned with an Aperio ScanScope (Leica Biosystems) at ×40 (0.25 μm/pixel). Using Aperio ImageScope software, the AR-V7–stained TMA slides were annotated to create regions of interest (ROIs) for analysis. Quantitative image analysis of the annotated ROIs was performed using Aperio Brightfield Image Analysis Toolbox software (Leica Biosystems). The analysis data for each TMA spot were extracted into Microsoft Excel for further analysis. The quantitative analysis data for each TMA spot included total numbers and percentages of nuclei (positive and negative), average positive intensity, average positive OD, and area of analysis. The intensity is a measurement of the light transmission, or brightness, of the positive staining in the nuclei and is logarithmically related to the OD. The OD is a measurement of absorbance and is linearly related to the amount of staining present. Automated scores for AR-V7 protein expression were reviewed and confirmed by a pathologist (L.D. True), and have been shown to correlate highly with manual scoring (Supplemental Figure 9 and refs. 84–86).

### AR mutation and copy number analysis

AR mutations and AR copy number were determined for CRPC patient biopsies as previously described (87).

### RNA-Seq analysis

#### UW CRPC cohort.

A set of 41 metastatic tumors from 24 men with CRPC were obtained through the University of Washington Prostate Cancer Donor Autopsy Program and used for transcript profiling by RNA-Seq, as described using frozen tissues (31, 88). RNA-Seq data are deposited in the Gene Expression Omnibus database under the accession number GSE118435. These tissues were from metastases where we had tissue available from the same block that had been used to spot the tissue microarray. Tissue microarray AR-V7 IHC scores were matched to mRNA samples by block ID. We then computed the Pearson correlation between AR-V7 expression (automated digital scoring) and gene mRNA expression (log_2_ counts per million), controlling for multiple testing using the cor.test and qvalue functions in R. There were 487 genes with *q* value less than 0.05, 407 of which correlated with higher expression in AR-V7–expressing tumors. This 407-gene signature was independently tested in a set of 21 CRPC biopsies (ICR/RMH cohort; see below) and 122 CRPC transcriptomes (SU2C/PCF cohort; see below). AR-FL and AR-V7 mRNA expression in spliced reads per million mapped reads for 41 CRPC transcriptomes from the UW cohort were calculated as previously described (89). Junction reads spanning the AR exon 3 to exon 4 junction were used to estimate AR-FL–specific expression, while reads spanning the AR exon 3 to cryptic exon 3 junction were used to estimate AR-V7–specific reads, normalized by total spliced reads (genome-wide) to correct for sequencing depth.

#### ICR/RMH cohort.

Twenty-one patients (from the SU2C/PCF consortium) with AR-V7 protein expression by IHC and RNA-Seq analysis from the same biopsy were used. Data from 21 transcriptomes generated by the International Stand Up To Cancer/Prostate Cancer Foundation (SU2C/PCF) Prostate Cancer Dream Team were downloaded and reanalyzed (3). Paired-end transcriptome sequencing reads were aligned to the human reference genome (GRCh37/hg19) using TopHat2 (version 2.0.7). Gene expression, fragments per kilobase of transcript per million mapped reads (FPKM), was calculated using Cufflinks (90). For those genes identified in the UW CRPC cohort, association between nuclear AR-V7 protein expression (IHC) and each gene mRNA expression (RNA-Seq) from the same biopsy was determined using Pearson correlation coefficient.

#### SU2C/PCF cohort.

Data from 122 CRPC transcriptomes generated by the International Stand Up To Cancer/Prostate Cancer Foundation (SU2C/PCF) Prostate Cancer Dream Team were downloaded and reanalyzed as described above. For those genes identified in the UW CRPC cohort, association between AR-V7 mRNA expression and each gene mRNA expression was determined using Pearson correlation coefficient.

### Pathway enrichment analysis

Of the 407 genes positively associated with higher AR-V7 levels with a *q* value less than 0.05 in the UW CRPC cohort (described above), 59 were found to be positively associated and significant with *P* less than 0.05 in either the ICR/RMH cohort or the SU2C/PCF cohort. The UpSetR R package was used to plot overlap between cohorts. Pathway overrepresentation analysis of the 407- and 59-gene sets was conducted using the Compute Overlaps tool with MSigDB v6.2 (H, Hallmark; CP, Canonical Pathways; C4, Computational Gene Sets; C5, GO; and C6, Oncogenic Pathways) (49, 50, 91).

### Statistics

All statistical analyses were conducted using Stata v13.1 or GraphPad Prism v6 and are indicated within all figures and tables. H scores (HSs) were reported as median values with interquartile range. For the ICR/RMH CSPC and CRPC cohort, Mann-Whitney test was used to compare differences in nuclear AR-V7 protein expression levels. Spearman’s rank correlation coefficient was used to determine the association between nuclear AR-V7 protein expression and timing of CRPC biopsy after the starting of AA or E therapy. Nonparametric equality-of-medians test was used to determine the difference in nuclear AR-V7 protein expression between metastatic sites. Wilcoxon matched-pairs signed-rank test was used to determine the difference in nuclear AR-V7 protein expression as VCaP mouse xenografts progressed from castration sensitive through castration resistant to AA/E resistant. Spearman’s rank correlation coefficient was used to determine associations between AR-FL and AR-V7 mRNA expression, total AR-V7 and total AR-FL protein expression, total AR-V7 protein expression and AR copy number, total AR-FL protein expression and AR copy number, nuclear AR-V7 and nuclear AR-FL protein expression, and OD and HS quantification for nuclear AR-V7 expression. Fligner-Killeen’s and Levene’s tests for homogeneity of variances between tumors and within tumors were performed in R using the fligner.test and leveneTest functions. Patients’ responses to AR-targeted therapy (AA or E) before and after chemotherapy and docetaxel were determined. For each therapy, PSA nadir was calculated as the lowest PSA level on therapy, and 12-week PSA response was calculated as the percentage change in PSA between the start of therapy (baseline) and 12 weeks of treatment (or closest available PSA reading). Time to PSA progression was defined as time from start of therapy to first PSA increase that is at least 25% and at least 2 μg/l above the PSA nadir. Time to clinical/radiological progression was defined as time from start of therapy to documented radiological progression or clinical progression (including change of therapy, addition of investigational medicinal product, or stopping of treatment). Overall survival was defined as time from start of therapy to date of death or last follow-up/contact. Patients’ baseline characteristics and clinical outcomes were compared by positive (nuclear AR-V7 HS >10) or negative (nuclear AR-V7 HS ≤10) AR-V7 status. Patients’ baseline characteristics were compared using Fisher’s exact test, Student’s *t* tests (2-tailed), and Wilcoxon rank-sum test as indicated. The 50% PSA nadir and 12-week 50% PSA response rates were compared using Fisher’s exact test. Nuclear AR-V7 expression by 50% PSA response rate was compared using Mann-Whitney *U* test. Median time to PSA progression, time to clinical/radiological progression, and overall survival were estimated using the Kaplan-Meier method. Association with AR-V7 status (positive vs. negative) was tested using univariable Cox proportional hazards models. For all statistical analysis, a *P* value less than 0.05 was considered to be statistically significant.

### Study approval

All animal studies were performed in accordance with Beth Israel Deaconess Medical Center (Boston, Massachusetts, USA) IACUC regulations (protocol 086-2016). All patients treated at the Royal Marsden NHS Foundation Trust had provided written informed consent and were enrolled in institutional protocols approved by the Royal Marsden NHS Foundation Trust Hospital (London, United Kingdom) ethics review committee (reference 04/Q0801/60). All procedures involving human subjects at the University of Washington (Seattle, Washington, USA) and Fred Hutchinson Cancer Research Center (Seattle, Washington, USA) were approved by the Institutional Review Board at those institutions.

## Author contributions

AS, IC, WY, CS, DNR, JWR, IF, CB, DD, GS, RR, TU, AN, JW, CM, SC, PSN, SPB, LDT, JSDB, and SRP designed the research studies. AS, IC, WY, CS, DNR, JWR, IF, CB, RR, TU, AN, JW, SC, and LDT conducted experiments and acquired data. AS, IC, WY, CS, DD, DNR, JWR, IF, CB, GS, TU, AN, JW, SC, PSN, SPB, LDT, JSDB, and SRP analyzed the data. AS, IC, WY, CS, DD, DNR, JWR, IF, CB, GS, RR, TU, AN, JW, CM, SC, JL, PSN, SPB, LDT, JSDB, and SRP wrote and critically reviewed the manuscript.

## Supplementary Material

Supplemental data

ICMJE disclosure forms

Supplemental Table 7

Supplemental Table 9

Supplemental Table 10

## Figures and Tables

**Table 1:**
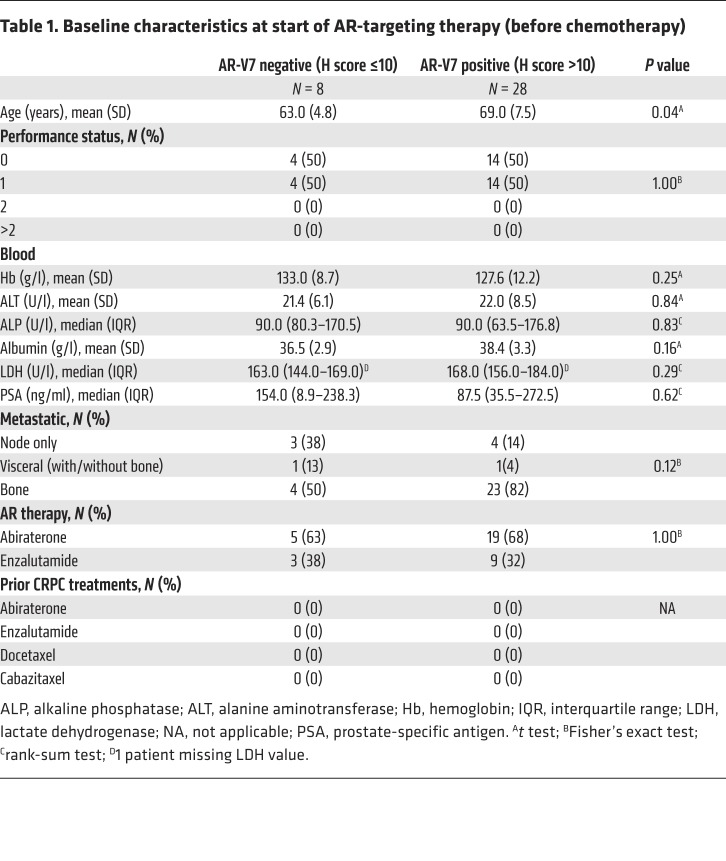
Baseline characteristics at start of AR-targeting therapy (before chemotherapy)

## Version 1 10/18/2018

In-Press Preview

## Version 2 11/26/2018

Electronic publication

## Version 3 12/13/2018

AR mutation and copy number analysis section added to Methods; Figure 5 edited; Reference 28 corrected; References 29 and 87 added; Minor typographical corrections made throughout the text.

## Version 4 01/02/2019

Print issue publication
