# Supplementary material for: Androgen receptor splice variant-7 expression emerges with castration resistance in prostate cancer
Source: J Clin Invest. 2018 Nov 26;129(1):192–208. doi: 10.1172/JCI122819 (PMC6307949; doi:10.1172/JCI122819)
Supplement: Supplemental data [file jci-129-122819-s116.pdf]

11. Supplementary figures and supplementary figure legends

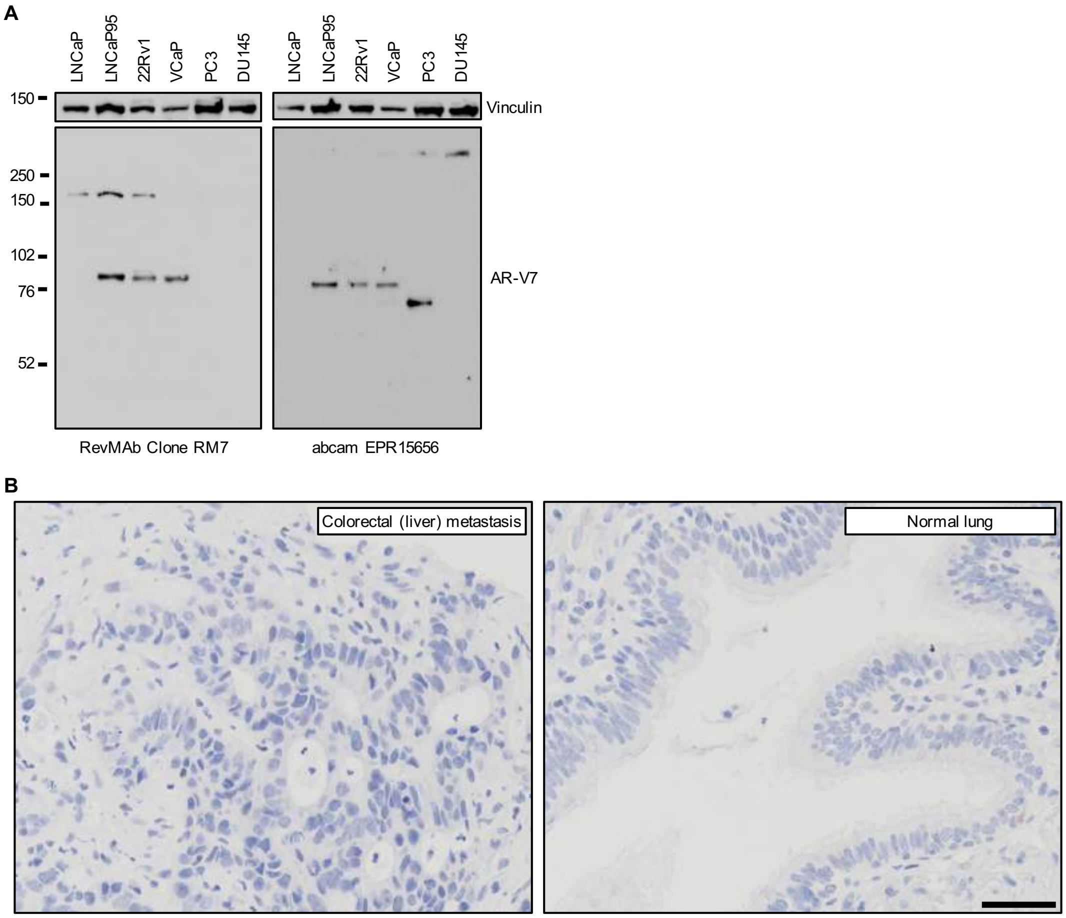

Supplementary Figure S1: Validation and optimization of novel AR-V7 antibody for immunohistochemistry.

(A) Western blot (short exposure) of AR-V7 positive (LNCaP95, 22Rv1 and VCaP) and negative (LNCaP, PC3 and DU145) PC cell lines using a novel recombinant rabbit monoclonal anti-AR-V7 antibody (Clone RM7) and previously reported anti-AR-V7 antibody (EPR15656). All cell lines except for LNCaP95 (10% charcoal stripped serum) were grown in 10% fetal bovine serum. **(B)** Micrographs of AR-V7 detection by IHC in colorectal liver metastasis and normal lung tissue known to be negative for AR-V7 RNA (magnification 200x; scale bar 50 µm) [30].

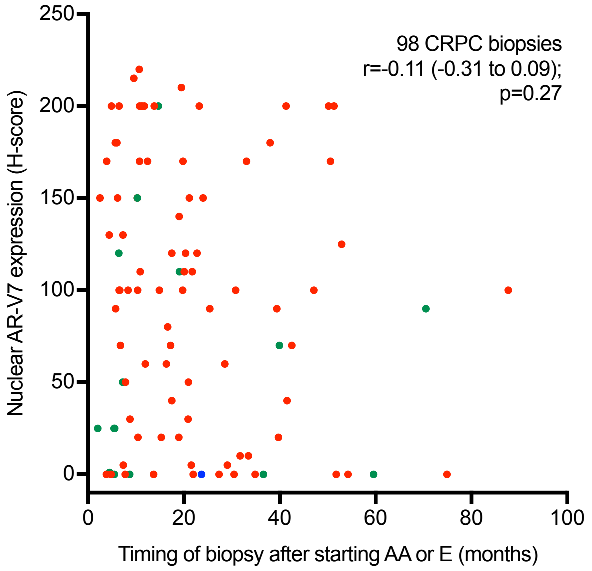

Supplementary Figure S2: Association between AR-V7 protein expression and timing of CRPC biopsies after starting abiraterone acetate or enzalutamide therapy.

Expression (H-score) of nuclear AR-V7 expression compared to timing (months) of CRPC biopsies (for 98 of 120 biopsies with treatment dates) after starting abiraterone acetate (AA) or enzalutamide (E) therapy. Those patients having completed therapy (red) and continuing therapy (green) at time of biopsy are shown. One AR null CRPC case with neuroendocrine features is shown (blue). Spearman's rank correlation is shown.

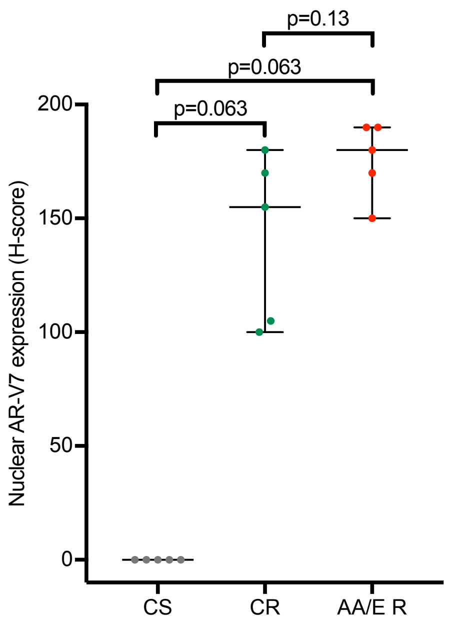

Supplementary Figure S3: Quantification of AR-V7 protein expression in VCaP mouse xenografts in response to therapy.

Expression (H-score) of nuclear AR-V7 in five VCaP mouse xenograft models as they progress from castration sensitive (CS; grey) through castration resistant (CR; green) to abiraterone acetate/enzalutamide resistant (AA/E R; red). Median HS and interquartile range is shown. p-value was calculated using Wilcoxon matched-pairs signed rank test.

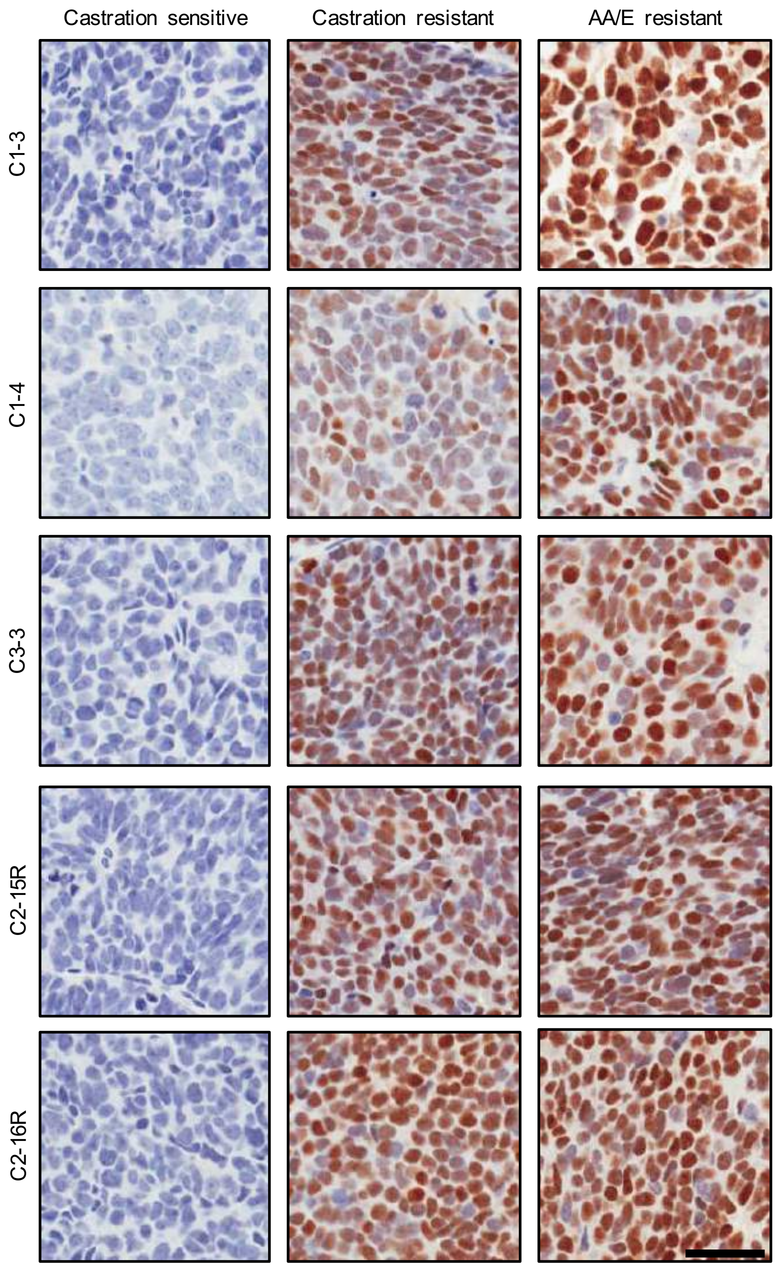

Supplementary Figure S4: Micrographs of AR-V7 protein expression in VCaP mouse xenografts in response to therapy.

Micrographs of nuclear AR-V7 expression in five VCaP mouse xenograft models as they progress from castration sensitive through castration resistant to abiraterone acetate (AA) and enzalutamide (E) resistant (magnification 200x; scale bar 50 μm).

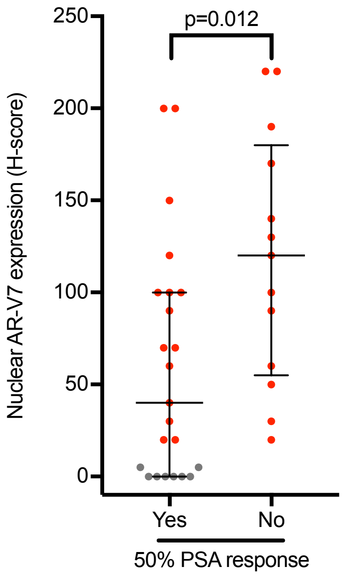

Supplementary Figure S5: Quantification of AR-V7 protein expression by 50% PSA response rate to AR targeting therapies (abiraterone acetate or enzalutamide) prior to chemotherapy in castration resistant prostate cancer.

Expression (H-score) of nuclear AR-V7 in castration resistant prostate cancer biopsies by 50% PSA response rate is shown. Median HS and interquartile range is shown. AR-V7 negative (H-score ≤ 10; grey) and AR-V7 positive (H-score > 10; red) are also shown. p-value was calculated using Mann-Whitney test.

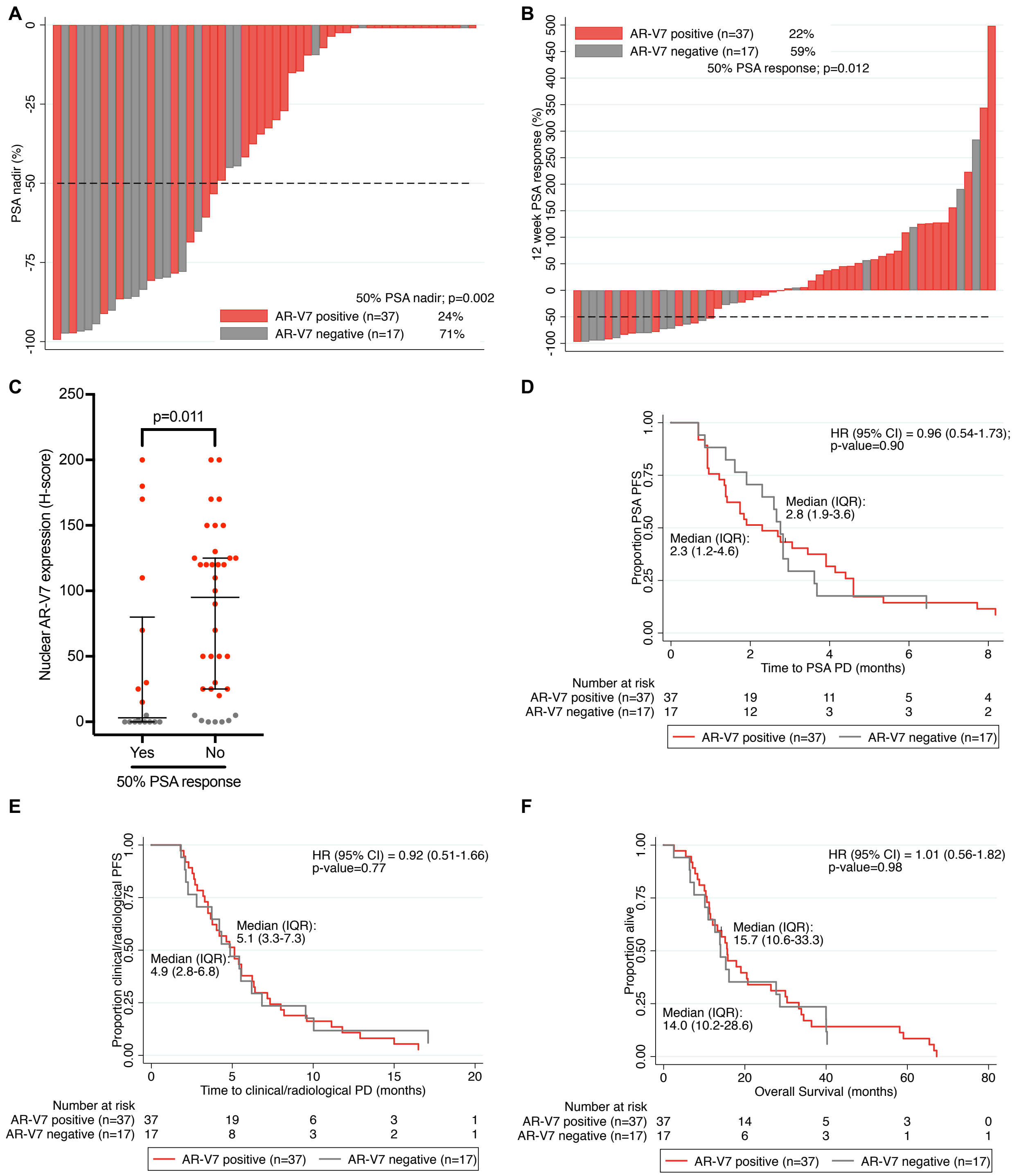

Supplementary Figure S6: AR-V7 status and response to AR targeting therapies (abiraterone acetate or enzalutamide) after chemotherapy in castration resistant prostate cancer.

Fifty-four patients received AR targeting therapies (abiraterone acetate or enzalutamide) after chemotherapy for CRPC. **(A)** Percentage PSA nadir on AR targeting therapies for

AR-V7 negative (H-score ≤ 10; grey) and AR-V7 positive (H-score > 10; red) CRPC patients is shown. 50% PSA nadir rate is shown. p-value was calculated using Fisher's exact test. **(B)** Percentage 12-week 50% PSA response rate on AR targeting therapies for AR-V7 negative (grey) and AR-V7 positive (red) CRPC patients is shown. Twelve-week 50% PSA response rate is shown. p-value was calculated using Fisher's exact test. **(C)** Expression (H-score) of nuclear AR-V7 in castration resistant prostate cancer biopsies by 50% PSA response rate is shown. Median HS and interquartile range is shown. AR-V7 negative (H-score ≤ 10; grey) and AR-V7 positive (H-score > 10; red) are also shown. p-value was calculated using Mann-Whitney test. **(D-F)** Kaplan-Meier curves show time to PSA progression (D), time to clinical/radiological progression (E) and overall survival (F) from starting AR targeting therapy. Hazard ratios (HR) with 95% confidence intervals (CI) are shown. p-value was calculated using univariate Cox proportional hazards model.

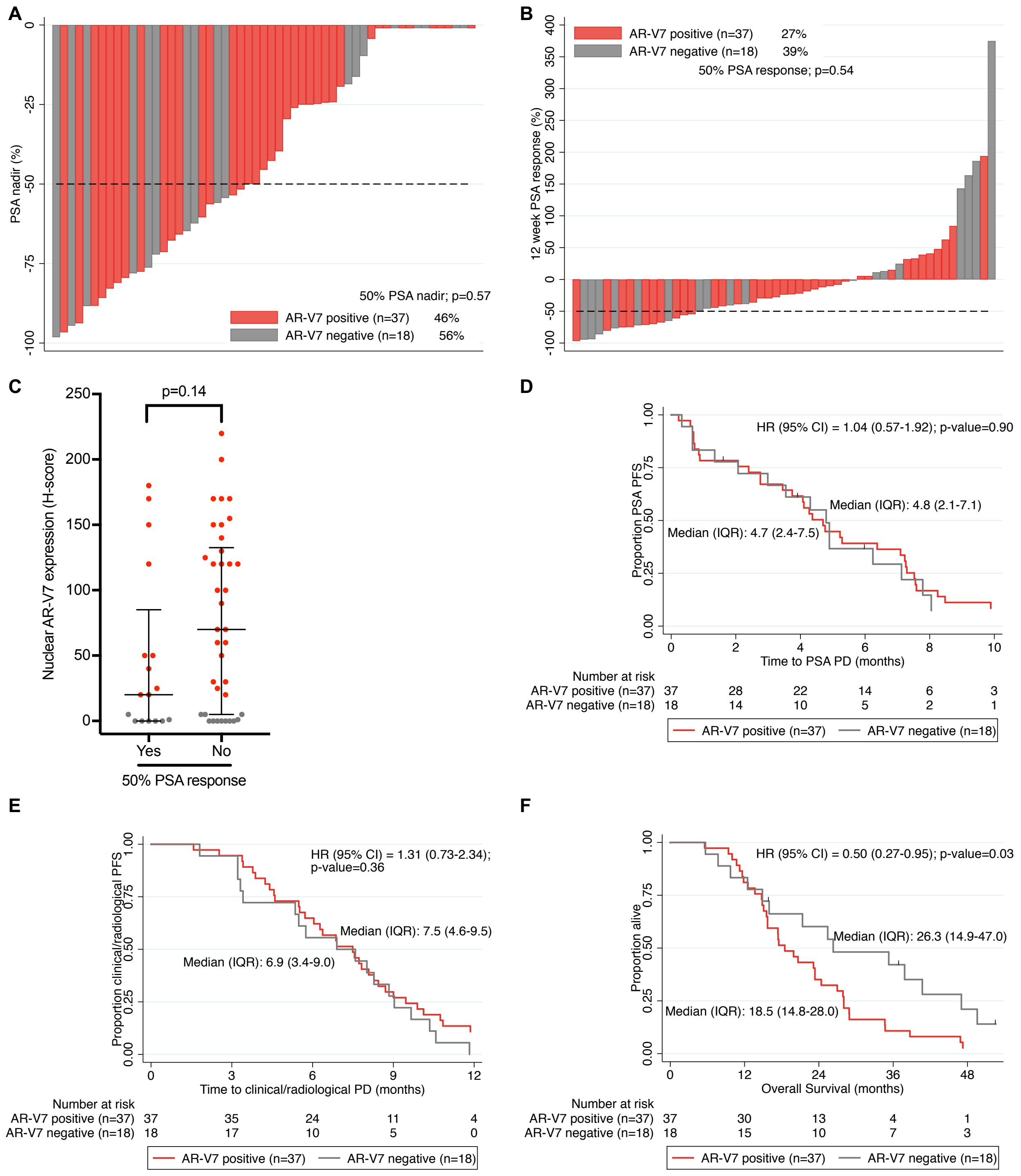

Supplementary Figure S7: AR-V7 status and response to docetaxel chemotherapy in castration resistant prostate cancer.

Fifty-five patients received docetaxel chemotherapy for CRPC. **(A)** Percentage PSA nadir on docetaxel chemotherapy for AR-V7 negative (H-score ≤ 10; grey) and AR-V7 positive (H-score > 10; red) CRPC patients is shown. 50% PSA nadir rate is shown. p-

value was calculated using Fisher's exact test. **(B)** Percentage 12-week 50% PSA response rate on docetaxel chemotherapy for AR-V7 negative (grey) and AR-V7 positive (red) CRPC patients is shown. Twelve-week 50% PSA response rate is shown. p-value was calculated using Fisher's exact test. **(C)** Expression (H-score) of nuclear AR-V7 in castration resistant prostate cancer biopsies by 50% PSA response rate is shown. Median HS and interquartile range is shown. AR-V7 negative (H-score ≤ 10; grey) and AR-V7 positive (H-score > 10; red) are also shown. p-value was calculated using Mann-Whitney test. **(D-F)** Kaplan-Meier curves show time to PSA progression (D), time to clinical/radiological progression (E) and overall survival (F) from starting docetaxel therapy. Hazard ratios (HR) with 95% confidence intervals (CI) are shown. p-value was calculated using univariate Cox proportional hazards model.

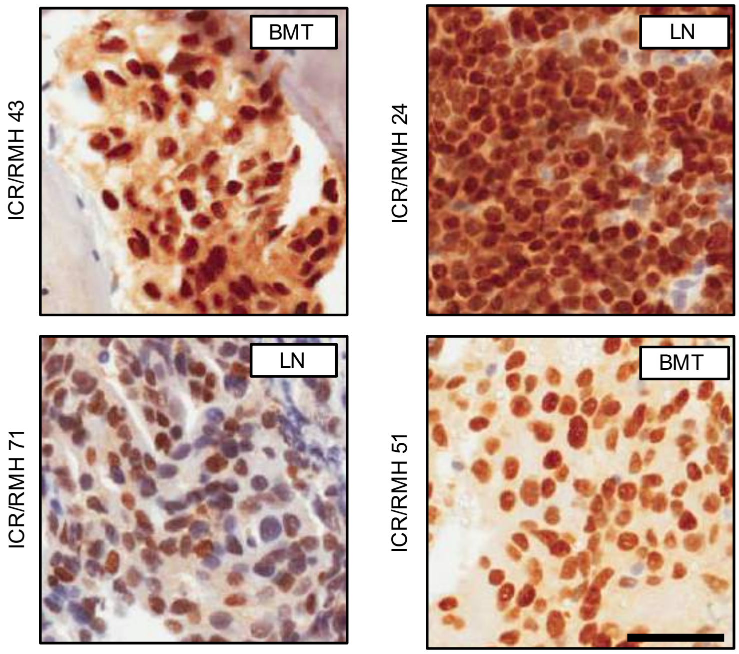

Supplementary Figure S8: AR-FL protein expression in advanced prostate cancer.

Representative micrographs of AR-FL (N-terminal domain) detection by immunohistochemistry (IHC) in four ICR/RMH patients with CRPC biopsies (magnification 200x; scale bar 50 μm).

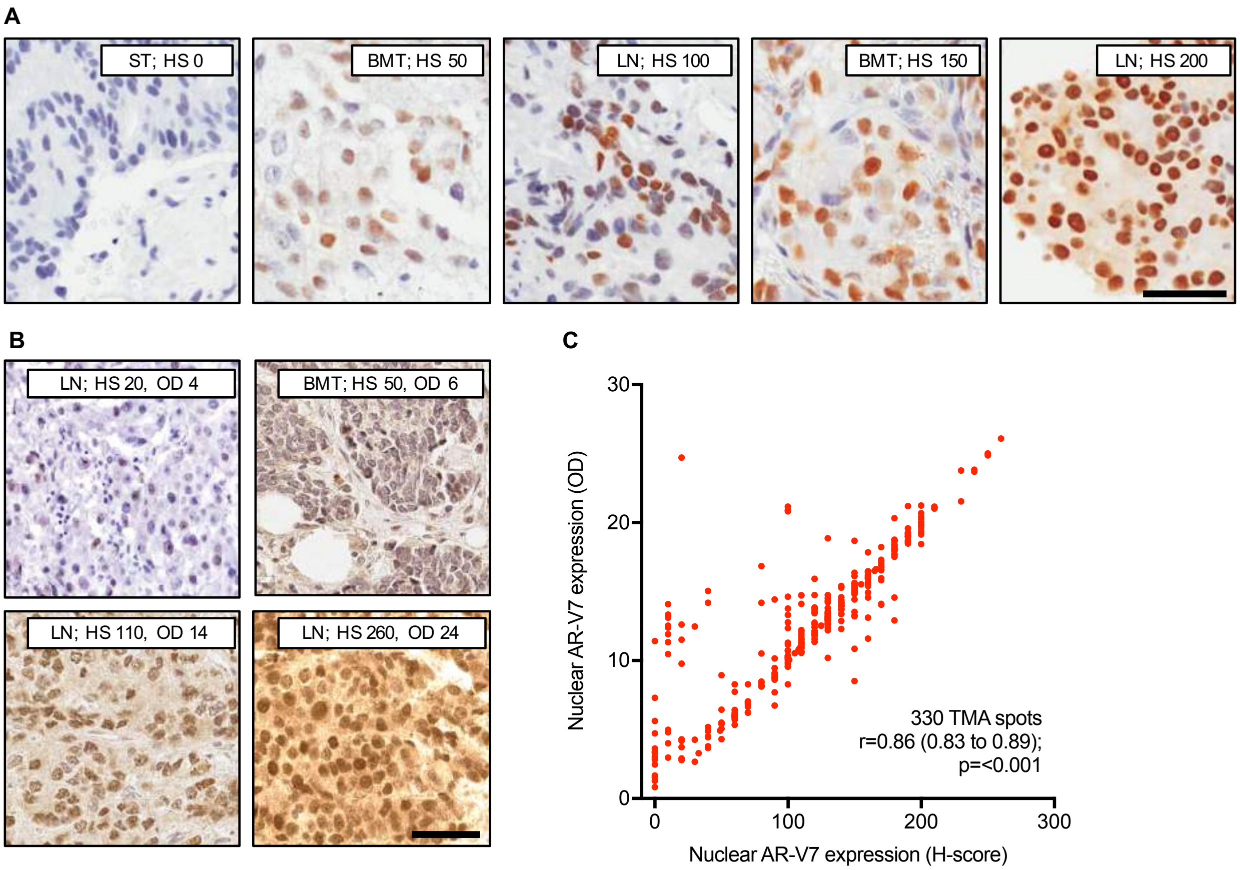

Supplementary Figure S9: Comparison of automated and manual quantification of AR-V7 expression from tissue biopsies of advanced prostate cancer.

(A) Representative micrographs of five CRPC biopsies from ICR/RMH patient cohort demonstrating varying levels of AR-V7 expression (measured by H-score; HS) are shown (magnification 200x; scale bar 50 μm). Biopsy site (Soft Tissue – ST, Bone Marrow Trephine – BMT, Lymph Node – LN) is shown. **(B)** Representative micrographs of four CRPC biopsies from UW patient cohort demonstrating varying levels of AR-V7 expression (measured by optical density; OD and HS) are shown (magnification 200x; scale bar 50 μm). Biopsy site (Bone Marrow Trephine – BMT, Lymph Node – LN) is shown. **(C)** Comparison between OD and HS for three TMA spots per metastasis from 110 (of 133) CRPC biopsies in the UW CRPC cohort. Spearman's rank correlation is shown.

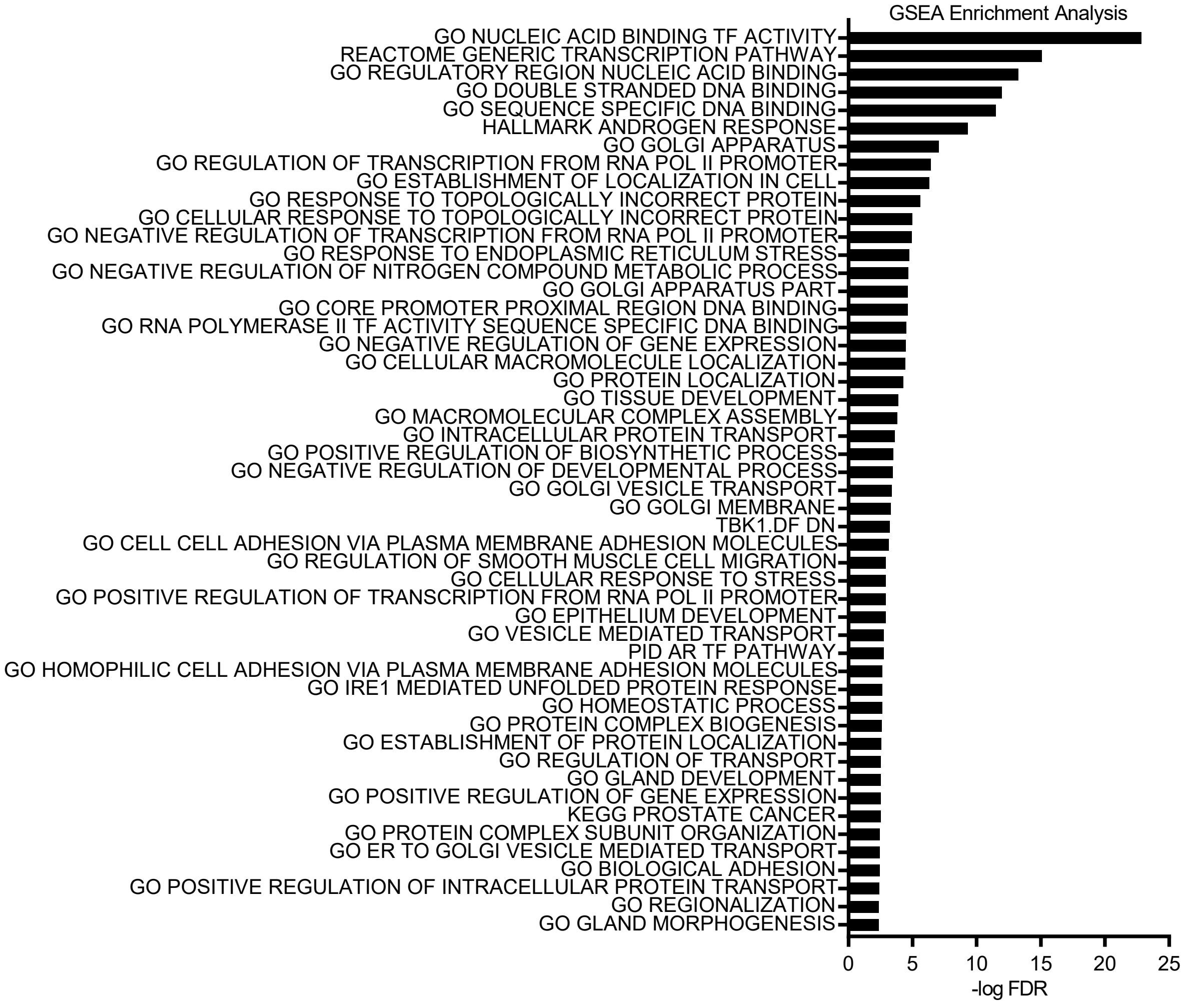

Supplementary Figure S10: Pathway analysis of 407 genes that associate with AR-V7 protein expression in UW CRPC cohort.

Pathway over-representation analysis using MSigDBv6.2 (H – Hallmark, CP – Canonical Pathways, C4 – Computational Gene Sets, C5 – GO and C6 – Oncogenic Pathway) in the 407-upregulated gene set identified in the UW CRPC cohort. Pathways with FDR <0.005 are shown.

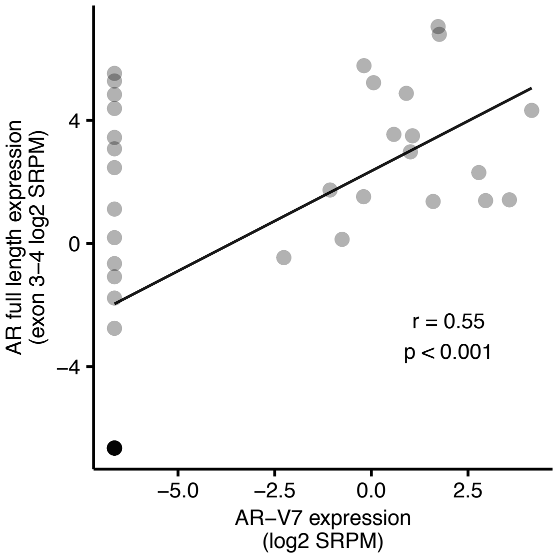

Supplementary Figure S11: AR-FL and AR-V7 mRNA expression UW CRPC cohort.

AR-FL and AR-V7 mRNA expression in spliced reads per million mapped reads (SRPM) for 41 transcriptomes from the UW CRPC cohort is shown with Pearson correlation. Gapped reads spanning exon 3 to exon 4 junction are used to estimate AR-FL specific expression, while reads spanning exon 3 to cryptic exon 3 junction are used to estimate AR-V7 specific reads, both normalized by total spliced reads (genome-wide) to correct for sequencing depth.

12. Supplementary Tables

	CSPC biopsies N=63
**Age, years** Mean (SD)	61.7 (7.52)
**Histology, N (%)** Adenocarcinoma	63 (100)
**Gleason, N (%)**	
≤7	18 (29)
>7	42 (67)
NR	3 (5)
**Presenting PSA, ng/mL** Median (IQR)	51.7 (20.3-145.0)^
**Stage at diagnosis, N (%)**	
T1	1 (2)
T2	1 (2)
T3	28 (44)
T4	11 (17)
NR	22 (35)
N0	17 (27)
N1	18 (29)
NR	28 (44)
M0	26 (41)
M1	25 (40)
NR	12 (19)
**Therapy intent, N (%)**	
Curative	27 (43)
Palliative	36 (57)

Supplementary Table 1: ICR/RMH CSPC cohort baseline characteristics at prostate cancer diagnosis.

N – number, SD – standard deviation, IQR – interquartile range, NR – not recorded, PSA – prostate specific antigen

^6 patient without PSA values; two values >2000 (analyzed as 2000)

	CSPC biopsies N=128
**Age, years**<br>Mean (SD)	60 (8.21)
**Histology, N (%)**<br>Adenocarcinoma	128 (100)
**Gleason, N (%)**	
≤7	39 (30)
>7	86 (70)
**Presenting PSA, ng/mL**<br>Median (IQR)	6.3 (3.3-67.0)
**Stage at diagnosis, N (%)**	
T1	0 (0)
T2	110 (86)
T3	17 (13)
T4	1 (1)
N0	125 (98)
N1	3 (2)
M0	128 (100)
M1	0 (0)
**Recurred, N (%)**	64 (50)

Supplementary Table 2: UW CSPC cohort baseline characteristics at prostate cancer diagnosis.

N – number, SD – standard deviation, IQR – interquartile range, NR – not recorded, PSA – prostate specific antigen

	CRPC biopsies N=160
**Age, years**	
Median (IQR)	68.5 (63.9-73.1)
**Performance status, N (%)**	
0	31 (19)
1	117 (73)
2	12 (8)
>2	0 (0)
**Bloods**	
Hb (g/L), Mean (SD)	114.9 (15.5)
ALT (U/L), Median (IQR)	16.5 (13.0-25.8)
ALP (U/L), Median (IQR)	127.0 (72.3-332.5)
Albumin (g/L), Median (IQR)	35.0 (33.0-38.0)
LDH (U/L), Median (IQR)	204.5 (156.0-309.0)
PSA (ng/mL), Median (IQR)	230.5 (77.0-591.5)
**Biopsy site, N (%)**	
Lymph node	51 (32)
Bone marrow trephine	81 (51)
Liver	10 (6)
Other	18 (11)
**Metastatic disease, N (%)**	
Node only	19 (12)
Visceral (with/without bone)	34 (21)
Bone	107 (67)
**Prior CRPC treatments, N (%)**	
0	20 (13)
1	32 (20)
2	50 (31)
3	40 (25)
4	18 (11)

Supplementary Table 3: ICR/RMH CRPC cohort baseline characteristics at time of CRPC biopsy.

N – number, SD – standard deviation, IQR – interquartile range, Hb – hemoglobin, ALT – alanine aminotransferase, ALP – alkaline phosphatase, LDH – lactate dehydrogenase, PSA – prostate specific antigen

	AR-V7 negative (H-score ≤ 10) N=17	AR-V7 positive (H-score > 10) N=37	p value
**Age, years**			
Median (IQR)	66.5 (58.2-72.1)	69.9 (64.2-73.2)	0.15[3]
**Performance status, N (%)**			
0	3 (18)	13 (35)	0.33[2]
1	12 (71)	22 (60)	
2	2 (12)	2 (5)	
>2	0 (0)	0 (0)	
**Bloods**			
Hb (g/L), Mean (SD)	115.8 (16.7)	118.6 (15.1)	0.55[1]
ALT (U/L), Median (IQR)	19.0 (15.0-24.5)	17.0 (14.0-26.5)	0.61[3]
ALP(U/L), Median (IQR)	131.0 (79.0-448.0)	81.0 (64-188)	0.08[3]
Albumin (g/L), Median (IQR)	34.0 (32.0-39.0)	35.0 (34.0-37.5)	0.64[3]
LDH (U/L), Median (IQR)	205.0 (152.0-327.5)^	181.0 (150.0-241.0)^^	0.36[3]
PSA (ng/mL), Median (IQR)	141.0 (53.0-254.0)*	207.0 (76.0-439.0)**	0.27[3]
**Metastatic, N (%)**			
Node only	1 (6)	7 (19)	0.41[2]
Visceral (with/without bone)	4 (24)	5 (14)	
Bone	12 (71)	25 (68)	
**AR therapy N (%)**			
Abiraterone	14 (82)	32 (87)	0.70[2]
Enzalutamide	3 (38)	5 (14)	
**Prior CRPC treatments, N (%)**			
Abiraterone	0 (0)	0 (0)	NA
Enzalutamide	0 (0)	0 (0)	NA
Docetaxel	17 (100)	37 (100)	1.00[2]
Cabazitaxel	2 (12)	1 (3)	0.22[2]

Supplementary Table 4: Baseline characteristics at time of starting AR targeting therapy (post-chemotherapy).

N – number, SD – standard deviation, IQR – interquartile range, Hb – hemoglobin, ALT – alanine aminotransferase, ALP – alkaline phosphatase, LDH – lactate dehydrogenase, PSA – prostate specific antigen

^1 patient missing LDH value
^^2 patients missing LDH value
*2 patients received docetaxel for CSPC
**2 patients received docetaxel for CSPC

1 t-test
2 Fisher's exact test
3 Rank-sum test

	AR-V7 negative (H-score ≤ 10) N=18	AR-V7 positive (H-score > 10) N=37	p value
**Age, years**			
Median (IQR)	66.4 (56.3-70.6)	69.0 (64.4-73.0)	0.07[1]
**Performance status, N (%)**			
0	5 (28)	13 (35)	
1	13 (72)	23 (62)	0.84[2]
2	0 (0)	1 (3)	
>2	0 (0)	0 (0)	
**Bloods**			
Hb (g/L), Mean (SD)	120.3 (13.4)	118.3 (13.8)	0.62[3]
ALT (U/L), Median (IQR)	17.0 (14.8-20.5)	21.0 (15.5-27.0)	0.16[1]
ALP(U/L), Median (IQR)	135.5 (78.8-259.8)	100.0 (70.0-216.5)	0.27[1]
Albumin (g/L), Mean (SD)	35.1 (4.3)	36.3 (3.6)	0.28[3]
LDH (U/L), Median (IQR)	206.5 (181.5-237.5)^	195.0 (148.0-261.0)^^	0.87[1]
PSA (ng/mL), Median (IQR)	126.0 (20.3-706.0)	116.0 (46.5-427.5)	0.72[1]
**Metastatic, N (%)**			
Node only	2 (11)	3 (8)	
Visceral (with/without bone)	4 (22)	4 (11)	0.48[2]
Bone	12 (67)	30 (81)	
**Prior CRPC treatments, N (%)**			
Abiraterone	5 (28)	11 (30)	1.00[2]
Enzalutamide	3 (17)	5 (14)	1.00[2]
Docetaxel	0 (0)	0 (0)	NA
Cabazitaxel	0 (0)	0 (0)	NA

Supplementary Table 5: Baseline characteristics at time of staring docetaxel chemotherapy.

N – number, SD – standard deviation, IQR – interquartile range, Hb – hemoglobin, ALT – alanine aminotransferase, ALP – alkaline phosphatase, LDH – lactate dehydrogenase, PSA – prostate specific antigen

^2 patient missing LDH value
^^2 patients missing LDH value

1 Rank-sum test
2 Fisher's exact test
3 t-test

	CRPC biopsies N=34 (133 biopsies)
**Age, years**	
Mean at diagnosis (SD)	62.0 (8.4)
Mean at death (SD)	68.0 (15.0)
**PSA, ng/mL**	
Mean at diagnosis (SD)	107.0 (259.0)
Mean at death (SD)	1205.0 (2642.0)
**Time from diagnosis to death, years**	
Mean (SD)	6.88 (5.39)
**Gleason, N (%)**	
<7	1 (2)
7	8 (24)
8-10	25 (74)
**Metastatic sites, N (%)**	
Visceral	33 (97)
Bone	34 (100)
**CRPC treatments, N (%)**	
Abiraterone	24 (70)
Enzalutamide	16 (47)
Apalutamide	1 (31)
Taxane	27 (79)

Supplementary Table 6: UW CRPC cohort baseline characteristics at time of CRPC biopsy.

N – number, SD – standard deviation, PSA – prostate specific antigen

Supplementary Table 7: Pathway analysis of 407 genes that associate with AR-V7 protein expression in UW CRPC cohort.

[See uploaded Excel file]

	CRPC biopsies N=21
**Age, years**	
Mean (SD)	69.3 (6.7)
**Performance status, N (%)**	
0	4 (19)
1	14 (67)
2	3 (14)
>2	0 (0)
**Bloods**	
Hb (g/L), Mean (SD)	118.5 (15.9)
ALT (U/L), Median (IQR)	19.0 (14.0-29.0)
ALP (U/L), Median (IQR)	120.0 (78.0-273.5)
Albumin (g/L), Median (IQR)	34.0 (32.0-36.5)
LDH (U/L), Median (IQR)	185.0 (159.5-233.5)^
PSA (ng/mL), Median (IQR)	260.0 (61.5-673.5)^^
**Biopsy site, N (%)**	
Lymph node	8 (38)
Bone marrow trephine	7 (33)
Liver	3 (5)
Other	3 (5)
**Metastatic disease, N (%)**	
Node only	1 (5)
Visceral (with/without bone)	7 (33)
Bone	13 (62)
**Prior CRPC treatments, N (%)**	
Abiraterone	16 (76)
Enzalutamide	7 (33)
Docetaxel	18 (86)
Cabazitaxel	9 (43)

Supplementary Table 8: Baseline characteristics of 21 ICR/RMH CRPC biopsies with nuclear AR-V7 protein expression and RNA-seq.

N – number, SD – standard deviation, IQR – interquartile range, Hb – hemoglobin, ALT – alanine aminotransferase, ALP – alkaline phosphatase, LDH – lactate dehydrogenase, PSA – prostate specific antigen

^1 patient missing LDH value
^^1 patient PSA value <0.1; analysed as 0.1

Supplementary Table 9: AR-V7 associated genes identified in UW CRPC cohort and validated using independent CRPC patient cohorts.

[See uploaded Excel file]

Supplementary Table 10: Pathway analysis of 59 independently validated genes that associate with AR-V7 protein expression in CRPC.

[See uploaded Excel file]

STROBE Statement—Checklist of items that should be included in reports of ***cohort studies***

****Please note that this STROBE Statement refers to the clinical correlations studies performed****

	Item No	Recommendation
**Title and abstract**	1	(*a*) **Androgen receptor splice variant-7 expression emerges with castration resistance in prostate cancer**
		(*b*) **Background:** Liquid biopsies have demonstrated that the constitutively active androgen receptor splice variant-7 (AR-V7) associates with reduced response and overall survival (OS) from endocrine therapies in castration resistant prostate cancer (CRPC). However, these studies provide little information pertaining to AR-V7 expression in prostate cancer (PC) tissue.<br><br>**Methods:** Following generation and validation of a novel AR-V7 antibody for immunohistochemistry, AR-V7 protein expression was determined for 358 primary prostate samples and 293 metastatic biopsies. Associations with disease progression, full length AR (AR-FL) expression, response to therapy, and gene expression was determined.<br><br>**Results:** We demonstrate that AR-V7 protein is rarely expressed (<1%) in primary PC but is frequently detected (75% of cases) following androgen deprivation therapy, with further significant (p=0.020) increase in expression following abiraterone acetate or enzalutamide therapy. In CRPC, AR-V7 expression is predominantly (94% of cases) nuclear and correlates with AR-FL expression (p=<0.001) and AR copy number (p=0.026). However, dissociation of expression is observed suggesting mRNA splicing remains crucial for AR-V7 generation. AR-V7 expression is heterogeneous between different metastases from a patient although AR-V7 expression is similar within a metastasis. Moreover, AR-V7 expression correlates with a unique 59-gene signature in CRPC, including HOXB13, a critical co-regulator of AR-V7 function. Finally, AR-V7 negative disease associates with better PSA responses (100% vs 54%; p=0.03) and OS (74.3 vs 25.2mo, HR 0.23 [0.07-0.79], p=0.02) from endocrine therapies (pre-chemotherapy).<br><br>**Conclusion:** This study provides impetus to develop therapies that abrogate AR-V7 signaling to improve our understanding of AR-V7 biology, and to confirm its clinical significance.
**Introduction**		
Background/rationale	2	Liquid biopsies have demonstrated that the constitutively active androgen receptor splice variant-7 (AR-V7) associates with reduced response and overall survival (OS) from endocrine therapies in castration resistant prostate cancer (CRPC). However, these studies provide little information pertaining to AR-V7 expression in prostate cancer (PC) tissue.
Objectives	3	Determine expression of nuclear AR-V7 in metastatic CRPC tissue biopsies and correlation with response to abiraterone acetate or enzalutamide (pre- and post-chemotherapy) and docetaxel therapy for CRPC.
**Methods**		
Study design	4	Observational cohort study of patients treated with abiraterone acetate or enzalutamide

		and docetaxel for CRPC with fully evaluable response data (50% PSA response, time to PSA progression, time to clinical/radiological progression and overall survival). All clinical data were collected retrospectively from electronic hospital records.
Setting	5	Patients were treated with abiraterone acetate or enzalutamide and docetaxel for CRPC at The Royal Marsden Hospital Foundation Trust.
Participants	6	(*a*) Please see **Figure 2**. Patients treated with abiraterone acetate or enzalutamide (pre- [n=36] and post-chemotherapy [n=54]) and docetaxel [55] for CRPC at The Royal Marsden Hospital Foundation Trust with fully evaluable response data (50% PSA response, time to PSA progression, time to clinical/radiological progression and overall survival).
		(*b*) Non-applicable
Variables	7	PSA Nadir, 50% PSA response, time to PSA progression, time to radiological/clinical progression and overall survival from starting abiraterone acetate or enzalutamide (pre- and post-chemotherapy) and docetaxel for CRPC by AR-V7 status was determined.
Data sources/ measurement	8*	Clinical data were obtained from electronic hospital records.
Bias	9	AR-V7 analysis was determined by a pathologist (DNR) blinded to clinical information and all statistical analysis was performed independently by statistician (DD).
Study size	10	Exploratory analysed performed on all patients that were fully evaluable (as described above; see **Figure 2**)
Quantitative variables	11	Patients baseline characteristics and clinical outcomes were compared by positive (nuclear AR-V7 HS > 10) or negative (nuclear AR-V7 HS ≤ 10) AR-V7 status.
Statistical methods	12	All statistical analyses were conducted using Stata v13.1 or GraphPad Prism v6 and are indicated within all figures and tables. H-scores (HS) were reported as median values with interquartile range (IQR). For ICR/RMH CSPC and CRPC cohort, Mann-Whitney test was used to compare differences in nuclear AR-V7 protein expression levels. Spearman's rank correlation coefficient was used to determine the association between nuclear AR-V7 protein expression and timing of CRPC biopsy after starting AA or E therapy. Nonparametric equality-of-medians test was used to determine the difference in nuclear AR-V7 protein expression between metastatic sites. Wilcoxon matched-pairs signed rank test was used to determine the difference between nuclear AR-V7 protein expression as VCaP mouse xenografts progressed from castration sensitive through castration resistant to AA/E resistance. Spearman's rank correlation coefficient was used to determine associations between AR-FL and AR-V7 mRNA expression, total AR-V7 and total AR-FL protein expression, total AR-V7 protein expression and AR copy number, total AR-FL protein expression and AR copy number, nuclear AR-V7 and nuclear AR-FL protein expression, and optical density and HS quantification for nuclear AR-V7 expression. Fligner-Killeen and Levene's tests for homogeneity of variances between-tumors and within-tumors were performed in R using the fligner.test and leveneTest functions. Patients response to AR targeted therapy (AA or E) pre- and post-chemotherapy, and docetaxel were determined. For each therapy, PSA nadir was calculated as the lowest PSA level on therapy and 12-week PSA response was calculated as the % change in PSA between the start of therapy (baseline) and 12-weeks treatment (or closest available PSA reading). Time to PSA progression was defined as time from start of therapy to first PSA increase that is ≥ 25% and ≥ 2μg/L above the PSA nadir. Time to clinical/radiological progression

		was defined as time from start of therapy to documented radiological progression or clinical progression (including change of therapy, addition of investigational medicinal product or stopping treatment). Overall survival was defined as time from start of therapy to date of death or last follow up/contact. Patients baseline characteristics and clinical outcomes were compared by positive (nuclear AR-V7 HS > 10) or negative (nuclear AR-V7 HS ≤ 10) AR-V7 status. Patient's baseline characteristics were compared using Fisher's exact test, Student's t-tests (2 tailed) and Wilcoxon rank-sum test as indicated. 50% PSA nadir and 12 week 50% PSA response rates were compared using Fisher's exact test. The difference between nuclear AR-V7 expression by 50% PSA response rate was compared using Mann-Whitney test. Median time to PSA progression, time to clinical/radiological progression and overall survival were estimated using the Kaplan-Meier method. Association with AR-V7 status (positive vs negative) was tested using univariable Cox proportional hazards models. For all statistical analysis, a p-value of less than 0.05 was considered to be statistically significant.
**Results**		
Participants	13*	(a) Patients treated with abiraterone acetate or enzalutamide (pre- [n=36] and post-chemotherapy [n=54]) and docetaxel [55] for CRPC.
		(b) Patients excluded did not have fully evaluable response data to abiraterone acetate or Enzalutamide and docetaxel.
		(c) Please see **Figure 2**
Descriptive data	14*	(a) Please see **Table 1** (abiraterone acetate or enzalutamide pre-chemotherapy), Supplementary **Table 4** (abiraterone acetate or enzalutamide post-chemotherapy) and Supplementary **Table 5** (docetaxel) for all baseline characteristics when starting therapy.
		(b) Any data missing is detailed in each of the tables above.
		(c) All patients were followed up to date of death or last follow up/contact.
Outcome data	15*	All outcome events are reported in **Figure 4** (abiraterone acetate or enzalutamide pre-chemotherapy)**, Supplementary Figure S6** (abiraterone acetate or enzalutamide post-chemotherapy) and **Supplementary Figure S7 (**docetaxel**).**
Main results	16	*(a)* **Abiraterone acetate or enzalutamide (pre-chemotherapy)**<br>Thirty-six patients received AA or E for CRPC prior to chemotherapy and had fully evaluable response data (**Figure 2**). Patients negative for AR-V7 (n=8) had a greater prostate specific antigen (PSA) 50% nadir (100 vs 68%, p=0.16) and PSA 50% response rate (100 vs 54%, p=0.03) than AR-V7 positive patients (n=28) (**Figure 4A-B**). Furthermore; AR-V7 negative patients had a longer time to PSA progression (11.5 vs 4.8 months (mo), hazard ratio (HR) 0.33 [0.14-0.81], p=0.02), longer time to clinical/radiological progression (13.9 vs 7.2 mo, HR 0.47 [0.20-1.10], p=0.08) and improved overall survival (74.3 vs 25.2mo, HR 0.23 [0.07-0.79], p=0.02) (**Figure 4C-E**).<br><br>**Abiraterone acetate or enzalutamide (post-chemotherapy)**<br>Fifty-four patients received AA or E for CRPC after chemotherapy and had fully evaluable response data (**Figure 2**). Patients negative for AR-V7 (n=17) had a significantly greater PSA 50% nadir (71 vs 24%; p=0.002) and PSA 50% response rate (59 vs 22%; p=0.012) than those positive for AR-V7 (n=37) (**Supplementary Figure S6A-B**). Interestingly, despite these significant differences in response rates, there was no significant difference in time to PSA progression (2.8 vs 2.3 mo, HR 0.96 [0.54-1.73], p=0.90), time to clinical/radiological progression (4.9 vs 5.1 mo, HR

		0.92 [0.51-1.66], p=0.77) or overall survival (14.0 vs 15.7 mo, HR 1.01 [0.56-1.82], p=0.98) (**Supplementary Figure S6D-F**).<br><br>**Docetaxel chemotherapy**<br>Fifty-five patients were treated with docetaxel chemotherapy for CRPC and had fully evaluable response data (**Figure 2**). In contrast to AR targeting therapies, there was no difference in PSA 50% nadir (56 vs 46%, p=0.57) and PSA 50% response rate (39 vs 27%, p=0.54) between AR-V7 negative (n=18) and positive patients (n=37) (**Supplementary Figure S7A-B**). Consistent with this, there was no significant difference in time to PSA progression (4.8 vs 4.7 mo, HR 1.04 [0.57-1.92], p=0.90) and time to clinical/radiological progression (6.9 vs 7.5 mo, HR 1.31 [0.73-2.34], p=0.36) (**Supplementary Figure S7D-E**). However, AR-V7 negative patients had improved overall survival (26.3 vs 18.5 mo, HR 0.50 [0.27-0.95], p=0.03) compared to AR-V7 positive patients (**Supplementary Figure S7F**).
		(*b*) Patients baseline characteristics and clinical outcomes were compared by positive (nuclear AR-V7 HS > 10) or negative (nuclear AR-V7 HS ≤ 10) AR-V7 status.
		(*c*) Non-applicable
Other analyses	17	Non-applicable
**Discussion**		
Key results	18	Taken together; these data confirm that AR-V7 is an important indicator of sensitivity to AR targeted therapies but not docetaxel treatment in CRPC.
Limitations	19	Retrospectively collected clinical data is the main limitation of this study.
Interpretation	20	Similar to AR-V7 expression determined by liquid biopsy; AR-V7 expression from direct metastatic CRPC biopsies demonstrates its role as a marker of resistance to AR targeting therapies.
Generalisability	21	Our results are in line with other studies that have utilised liquid biopsies that measured AR-V7 mRNA and protein from circulating tumour cells and identified it as a marker of resistance to AR targeting therapies.
**Other information**		
Funding	22	Work at University of Washington and in the Plymate and Nelson labs is supported by the Department of Defense Prostate Cancer Research Program (W81XWH-14-2-0183, W81XWH-12-PCRP-TIA, W81XWH-15-1-0430, and W81XWH-13-2-0070), the Pacific Northwest Prostate Cancer SPORE (P50CA97186), the Institute for Prostate Cancer Research (IPCR), Veterans Affairs Research Program, NIH/National Cancer Institute (P01CA163227), and the Prostate Cancer Foundation. Work in the de Bono laboratory was supported by funding from the Movember Foundation, Prostate Cancer UK, US Department of Defense, the Prostate Cancer Foundation, Stand Up To Cancer, Cancer Research UK, and the UK Department of Health through an Experimental Cancer Medicine Centre grant. AS is supported by the Medical Research Council, the Academy of Medical Sciences, Prostate Cancer UK and the Prostate Cancer Foundation.

*Give information separately for exposed and unexposed groups.

Note: An Explanation and Elaboration article discusses each checklist item and gives methodological background and published examples of transparent reporting. The STROBE checklist is best used in conjunction with this article (freely available on the Web sites of PLoS Medicine at http://www.plosmedicine.org/, Annals of Internal Medicine at

http://www.annals.org/, and Epidemiology at http://www.epidem.com/). Information on the STROBE Initiative is available at http://www.strobe-statement.org.
